# *Listeria monocytogenes* switches from dissemination to persistence by adopting a vacuolar lifestyle in epithelial cells

**DOI:** 10.1371/journal.ppat.1006734

**Published:** 2017-11-30

**Authors:** Mounia Kortebi, Eliane Milohanic, Gabriel Mitchell, Christine Péchoux, Marie-Christine Prevost, Pascale Cossart, Hélène Bierne

**Affiliations:** 1 Micalis Institute, Inra, AgroParisTech, Université Paris-Saclay, Equipe Epigénétique et Microbiologie Cellulaire, Jouy-en-Josas, France; 2 Department of Molecular and Cell Biology, University of California, Berkeley, Berkeley, California, United States of America; 3 Unité GABI, Inra, AgroParisTech, Université Paris-Saclay, Plate-Forme MIMA2, Jouy-en-Josas, France; 4 Institut Pasteur, Ultrapole, Paris, France; 5 Institut Pasteur, Unité des interactions Bactéries-Cellules, Paris, France; 6 Inserm, U604, Paris, France; 7 Inra, USC2020, Paris, France; University of Pennsylvania, UNITED STATES

## Abstract

*Listeria monocytogenes* causes listeriosis, a foodborne disease that poses serious risks to fetuses, newborns and immunocompromised adults. This intracellular bacterial pathogen proliferates in the host cytosol and exploits the host actin polymerization machinery to spread from cell-to-cell and disseminate in the host. Here, we report that during several days of infection in human hepatocytes or trophoblast cells, *L*. *monocytogenes* switches from this active motile lifestyle to a stage of persistence in vacuoles. Upon intercellular spread, bacteria gradually stopped producing the actin-nucleating protein ActA and became trapped in lysosome-like vacuoles termed *Lis**teria*-Containing Vacuoles (LisCVs). Subpopulations of bacteria resisted degradation in LisCVs and entered a slow/non-replicative state. During the subculture of host cells harboring LisCVs, bacteria showed a capacity to cycle between the vacuolar and the actin-based motility stages. When ActA was absent, such as in Δ*actA* mutants, vacuolar bacteria parasitized host cells in the so-called “viable but non-culturable” state (VBNC), preventing their detection by conventional colony counting methods. The exposure of infected cells to high doses of gentamicin did not trigger the formation of LisCVs, but selected for vacuolar and VBNC bacteria. Together, these results reveal the ability of *L*. *monocytogenes* to enter a persistent state in a subset of epithelial cells, which may favor the asymptomatic carriage of this pathogen, lengthen the incubation period of listeriosis, and promote bacterial survival during antibiotic therapy.

## Author summary

*L*. *monocytogenes* is a model intracellular pathogen that replicates in the cytoplasm of mammalian cells and disseminate in the host using actin-based motility. Here, we reveal that *L*. *monocytogenes* changes its lifestyle and persists in lysosomal vacuoles during long-term infection of human hepatocytes and trophoblast cells. When the virulence factor ActA is not expressed, subpopulations of vacuolar bacteria enter a dormant viable but non-culturable (VBNC) state. This novel facet of the *L*. *monocytogenes* intracellular life could contribute to the asymptomatic carriage of this pathogen in epithelial tissues and render it tolerant to antibiotic therapy and undetectable by routine culture techniques.

## Introduction

Over the past three decades, the study of the facultative intracellular pathogen *L*. *monocytogenes* has provided important insights into infection biology, immunity and host cell biology [1–5]. *L*. *monocyt*ogenes is a Gram-positive bacterium that belongs to the category of pathogens that escape from an internalization vacuole and proliferate in the cytosol of mammalian cells [6–8]. Following phagocytosis or receptor-mediated endocytosis, bacteria disrupt the invasion vacuole and enter the cytoplasm, where they replicate and use the actin cytoskeleton to propel themselves from cell-to-cell, thereby escaping intracellular autophagy and extracellular humoral immune responses [4, 5, 9]. To achieve this cytosolic lifestyle, *Listeria* deploys virulence effectors that specifically control a repertoire of host molecules and hijack cellular processes [4, 5]. For instance, the invasion proteins InlA and InlB promote bacterial entry into non-phagocytic cells by interacting with the surface receptors E-cadherin and c-Met, respectively [10]; the cytolysin listeriolysin O (LLO) disrupts the internalization vacuole and triggers various host cell responses [11, 12]; the listerial locomotion factor ActA mimics the host's actin nucleation machinery to stimulate actin polymerization, thereby promoting bacterial motility and intercellular spreading [13]. ActA also protects cytosolic bacteria from autophagic recognition by masking the bacterial surface [14, 15].

Despite potent virulence factors and the capacity to invade a variety of cell types, *L*. *monocytogenes* causes a relatively rare disease. Invasive listeriosis is characterized by severe clinical manifestations (septicemia, meningitis and miscarriages) and a high fatality rate (20–30% of cases), but its incidence remains low (1 to 10 cases per one million individuals [16]). The proposed explanation for this low incidence is that cell-mediated immunity is highly protective in healthy individuals. Accordingly, conditions that weaken the immune system, such as pregnancy, aging or immunosuppressive treatments, predispose individuals to listerial infection. However, even in susceptible individuals, the incubation period of listeriosis may be long, particularly in pregnancy-associated cases for which it can last up to three months [17]. In addition, *L*. *monocytogenes* is ubiquitously distributed in the environment and exposure to this pathogen appears to be relatively common, with 1–5% of people shedding these bacteria in their feces without developing symptoms [18]. Together, these data suggest that colonization of humans by *L*. *monocytogenes* involves an asymptomatic silent phase.

Until now, only a few studies support the existence of asymptomatic *L*. *monocytogenes* reservoirs. Immunodeficient SCID mice develop a chronic *Listeria* infection, particularly in hepatic macrophages, in which bacteria reside within large vacuoles [19]. Spacious *Listeria*-containing vacuoles have also been described in murine bone-marrow derived and RAW 264.7 macrophages cultured *in vitro* [20, 21]. These vacuoles, termed Spacious *L**isteri**a*-containing phagosomes (SLAPs), are phagosomes that do not mature into phagolysosomes and thus derive from a subpopulation of bacteria that never enter the cytosol. SLAPs are proposed to constitute a niche for *L*. *monocytogenes* survival in the host. The bone marrow and gall bladder are also potential asymptomatic reservoirs of *L*. *monocytogenes*, as revealed by bioluminescence imaging studies in mice [22, 23].

However, all these observations were made in murine models. Although studying *Listeria* infection in the mouse has led to the discovery of most *L*. *monocytogenes* virulence factors [4], this model has some limitations since murine listeriosis does not recapitulate all the characteristics of human listeriosis. In the mouse, oral infection with *L*. *monocytogenes* is not very efficient and bacteria do not appear to have an elective tropism for the central nervous system and the fetoplacental unit. The low efficiency of *L*. *monocytogenes* in crossing murine epithelial barriers is in part due to the lack of interaction between the invasion protein InlA and mouse E-cadherin, impairing bacterial entry in murine epithelial cells [24]. In addition, differences in mucosal immunity between mice and humans [25] may change the way these species respond to *L*. *monocytogenes* infection. For instance, *L*. *monocytogenes* activates expression of IFN-λ in human hepatocytes [26] and modulates IFN-λ responses [27], but hepatocytes do not respond to IFN-λ in the mouse [28]. Such species-specific differences may prevent the characterization of some of the mechanisms involved in prolonged *L*. *monocytogenes* infection in humans, particularly in epithelial tissues.

Human cells grown *in vitro* can be used to model the behavior of this pathogen during a long-term infection. However, rapid multiplication and dissemination of *Listeria* induces cell death and detachment, thereby forming lytic plaques. Thus, *in vitro* infections with *L*. *monocytogenes* have been restricted to short time courses (usually from a few minutes to one day). Here, we have developed a new experimental protocol for studying *L*. *monocytogenes* during prolonged infections of non-phagocytic human cells and found that, in hepatocytes and trophoblast cells, bacteria cease to polymerize actin and are enclosed in vacuoles after the actin-dependent motility phase. This phenotypic switch is correlated with a decreased expression of ActA at the bacterial surface. In addition, Δ*actA* mutants enter a viable but non-culturable (VBNC) state during long-term infection. The formation of LisCVs could potentially enable the persistence of this pathogen in epithelial tissues.

## Results

### *L*. *monocytogenes* localizes to LAMP1-associated compartments during a prolonged infection of human hepatocytes and trophoblast cells

**Fig 1:**
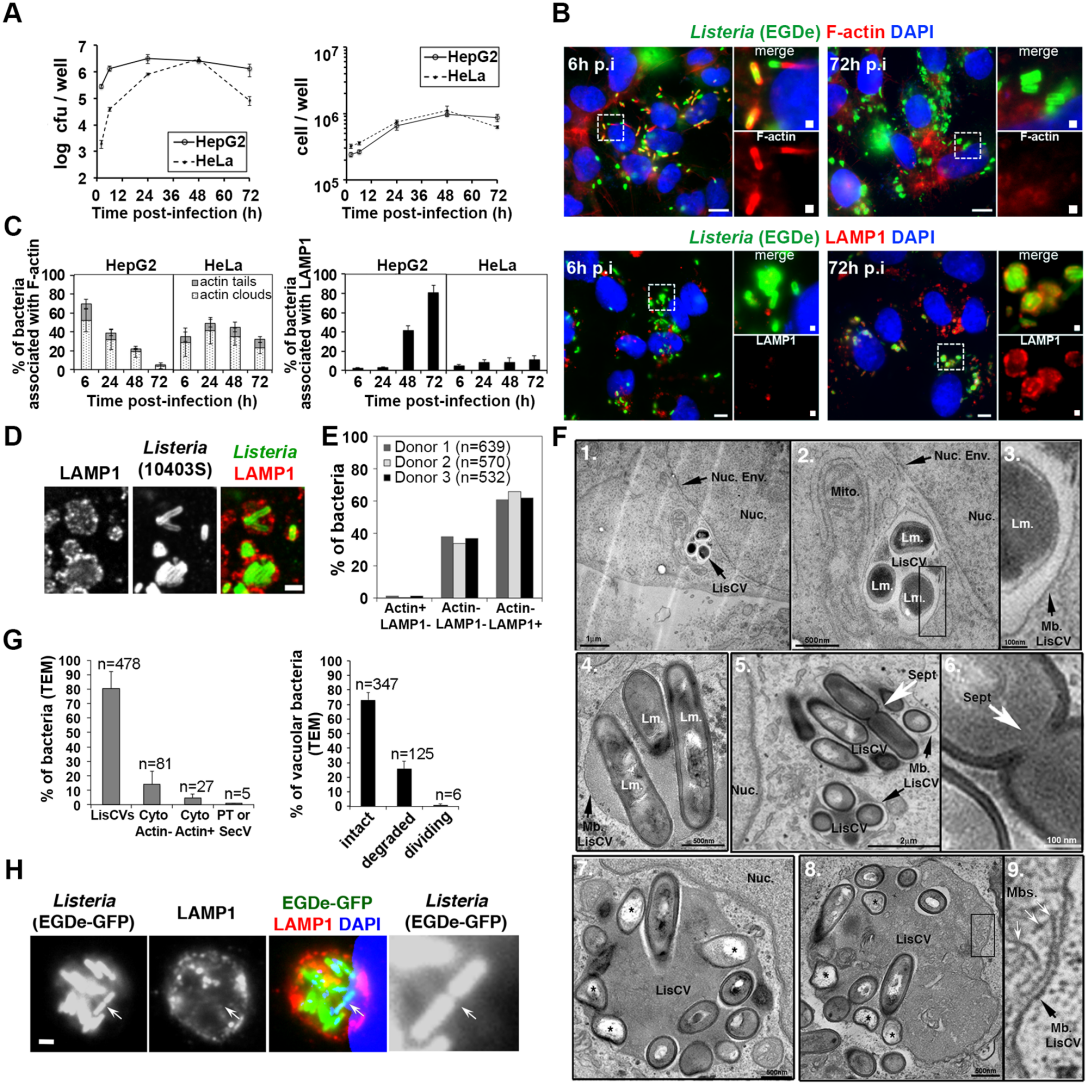
*L*. *monocytogenes* switches from actin-based motility to a vacuolar phase in human hepatocytes and trophoblast cells. **A-C** HepG2 or HeLa cells were infected with *L*. *monocytogenes* EGDe (MOI ~ 1–5), counted or lysed to determine bacterial intracellular loads by CFU counts, or processed for microscopy at the indicated time. **A**. Kinetics of bacterial and cell growth. Results are mean±SD of triplicate experiments. **B**. Micrographs of HepG2 cells infected for 6h (left panel) or 72h (right panel) with EGDe. Images are overlays of *Listeria* (green), F-actin or LAMP1 (red) and DAPI (blue) signals. Bars: 10 μm. High magnifications of the squared regions are shown beside with merged signals (on top) or single F-actin or LAMP1 signal (on bottom). Bars: 0.5 μm. **C**. Histograms of the percentage of intracellular bacteria associated with F-actin (left) or LAMP1 (right). At least 200 bacteria were examined per time-point. Results are mean±SD of triplicate experiments. **D**. Micrograph of primary human hepatocytes infected with *L*. *monocytogenes* 10403S for 72h (MOI ~ 5) and stained with LAMP1 (red in the overlay) and *Listeria* (green in the overlay) antibodies. Bar: 1 μm. **E**. Quantification of 10403S bacteria in different phenotypes at 72h p.i. in primary hepatocytes from three human donors. “n” indicates the number of scored bacteria. **F**. Ultrastructure of representative LisCVs at 72 h p.i. observed by TEM in HepG2 (images 1–3) or JEG3 cells (images 4–9). The nucleus (Nuc.), the nuclear envelope (Nuc. Env.), the membrane of the vacuole (Mb. LisCV), mitochondria (Mito.) and membranous structures (Mbs) are indicated. Images 1–3: a cluster of three *Listeria* (Lm.) sectioned along their short axis is enclosed within a single-membrane vacuole (LisCV). Three magnifications are shown: scale bars: 1 μm (1), 500nm (2) and 100nm (3). Image 4: three rod-shaped bacteria sectioned along their long axis within a LisCV. Bar: 500nm. Images 5–6: two LisCVs near the nucleus. The septum of a dividing bacterium is pointed with a white arrow (Sept.) and shown at a higher magnification in image 6. Bars: 2 μm and 100nm. Images 7–9: LisCVs in JEG3 cells containing clusters of bacteria, electron-dense heterogeneous materials and membranous structures (shown at a higher magnification in image 9). Altered bacteria are marked with *. Bars: 500 nm. **G**. Quantification of TEM-observed 10403S bacteria in JEG3 cells at 72h p.i. On the left, % of bacteria in LisCVs, in the cytosol (Cyto) either actin-free “Actin-” or polymerizing actin “Actin+”, and in protusions (PT) or secondary vacuoles (SecV) (also see S2 Fig). On the right, % of intact, degraded or dividing bacteria among vacuolar bacteria. Data are mean±SD of triplicate experiments. “n” indicate the total number of bacteria per category. **H**. Confocal micrographs of a LisCV. HepG2 cells were infected for 72h with *Listeria* EGDe-GFP (green) and processed for immunofluorescence with LAMP1 antibodies (red) and DAPI (blue). GFP stains the bacterial cytosol of bacteria in a LAMP1^+^ compartment. Bar: 1 μm. The arrow points the septum of a dividing bacterium, magnified in the black and white image.

In order to study long-term infection of epithelial cells by *L*. *monocytogenes*, the standard invasion protocol [29] was modified to reduce cytotoxicity. Bacterial inocula were prepared at stationary phase instead of exponential phase, which prevented rapid replication of bacteria at the onset of infection and increased the expression of invasion proteins InlA and InlB [30]. This enabled the use of a lower multiplicity of infection (MOI) and reduced cell damages caused by the bacterial toxin LLO. In addition, a high concentration of the membrane-impermeant antibiotic gentamicin was used for a short time (100 μg/ml for 10 minutes) to rapidly eliminate extracellular bacteria after the initial bacterial uptake. At this concentration, gentamicin does not enter the cells for at least 60 minutes [31]. Cells were then incubated in complete medium containing 25 μg/ml gentamicin, which eliminates extracellular bacteria and prevents cell reinfection by bacteria released from dead cells. Intracellular bacteria were enumerated by colony forming unit (CFU) at different time points for up to three days. Using this protocol with *L*. *monocytogenes* strain EGDe [32], marked differences were observed between the infection of HeLa cells and HepG2 hepatocytes (Fig 1A). Bacterial internalization was 100-fold higher in HepG2 cells than in HeLa cells, in agreement with the fact that both InlA and InlB mediate *L*. *monocytogenes* entry in HepG2 hepatocytes, whereas only the InlB pathway is functional in HeLa cells [33]. Following entry, bacterial loads sharply increased for 48h and then dramatically dropped in HeLa cells. In contrast, bacterial counts increased for 24h and only slightly decreased thereafter in HepG2 cells. No major differences in the number of infected cells per well were observed between these two cell lines (Fig 1A).

Microscopic examination of infected HepG2 cells revealed a striking decline in the number of bacteria associated with filamentous actin (F-actin) from 6h to 72h post-infection (p.i.) (Fig 1B). At the same time, there was an increase in the number of bacteria that colocalize with the late endosomal/lysosomal marker LAMP1 (Fig 1B). The percentage of bacteria associated with actin clouds or tails (Actin^+^) dropped from 70% to 5% between 6h and 72h p.i., while the percentage of LAMP1^+^ bacteria increased from 3% to 80% (Fig 1C). In contrast, the percentage of Actin^+^ bacteria did not change over time (30–50% of Actin^+^) and the percentage of LAMP1^+^ bacteria remained consistently low (5–10%) in HeLa cells (Fig 1C and S1A Fig). These results suggested that during long-term infection of HepG2 cells, bacteria stopped polymerizing actin and were incorporated into LAMP1^+^ vacuolar compartments.

Similar results were obtained in Huh7 hepatocytes (S1B Fig) as well as in JEG3 and BeWo trophoblast cells (S1C and S1D Fig), but not in HEK293 human embryonic kidney cells. This phenomenon was not strain-specific and was also observed with 10403S, the other widely used *L*. *monocytogenes* model strain [32], as well as with a listeriosis outbreak strain (CLIP63713, responsible for materno-fetal infections [34]) (S1C Fig): at 72h p.i in JEG3 cells, we quantified 80±5% of 10403S and 92±6% of CLIP63713 bacteria in LAMP1^+^ compartments. Thereafter, strains EGDe and 10403S were used for the study of this intracellular phenotype of *L*. *monocytogenes*.

### *L*. *monocytogenes* also localizes to LAMP1^+^ compartments after long-term infection of primary human hepatocytes

In order to rule out the possibility that this phenotypic switch was specific to transformed cell lines, experiments were performed in primary cells. Hepatocytes from the livers of three human donors were infected with strain 10403S. The efficiency of *L*. *monocytogenes* entry in primary hepatocytes was lower than in immortalized hepatocytes or HeLa cells (S1E Fig), as noticed for other primary cells [35], but bacterial loads were increased 200-fold after three days of infection (S1F Fig). Microscopic examination of hepatocytes infected for 72h with 10403S revealed that the majority of bacteria were enclosed in LAMP1^+^ compartments (Fig 1D and S1G Fig). More specifically, 63±3% of intracellular bacteria were positive for LAMP1, while most of the other bacteria were LAMP1-negative, but actin-free (36±2% of Actin^-^ LAMP1^-^). Only 1±0.5% of bacteria were associated with actin filaments (Actin^+^ LAMP1^-^) (Fig 1E). These results indicate that *L*. *monocytogenes* stops polymerizing actin and entered LAMP1^+^ compartment during long-term infection of primary human hepatocytes.

### Ultrastructure of the *Listeria*-containing vacuole

Transmission electron microscopy (TEM) analysis of cells infected for 72h revealed that LAMP1^+^ compartments were single-membrane bound vacuoles found in close proximity to the nucleus (Fig 1F, images 1–3). These *Lis**teria*-Containing Vacuoles (LisCVs) enclosed various amounts of bacteria (varying from one to twelve), which were rod-shaped in their longitudinal axis (Fig 1F, image 4). Only rare bacteria showed a division septum (Fig 1F, images 5–6). These vacuoles also contained damaged bacteria, electron-dense heterogeneous materials and membranous structures (Fig 1F, images 7–9). Of all intracellular bacteria observed with this technique (n = 591), 80% were in single-membrane vacuoles (mean ± SD of 3 ± 1 bacteria per vacuole). The other bacteria were cytosolic, either actin-free or associated with actin filaments, in membrane protrusions or within secondary vacuoles (Fig 1G). Examples of bacteria of each category are shown in S2 Fig. Importantly, 73% of vacuolar bacteria were morphologically intact, 1% exhibited a division septum, and 26% appeared degraded (Fig 1G). Of note, LisCVs were often close to compartments resembling electron-dense granular secondary lysosomes and to mitochondria (Fig 1F and S2 Fig).

Observation by confocal microscopy of GFP-expressing bacteria revealed staining of GFP in the bacterial cytosol, which indicates that the integrity of the envelope was preserved in most vacuolar bacteria (Fig 1H). As with TEM, rare bacteria had a division septum. Hence, vacuolar *Listeria* have the ability to divide, albeit slowly. This is consistent with the observation that LisCVs emerged after the phase of active cytosolic proliferation, which occurs on the first day of infection (Fig 1A).

### LisCVs are formed after the actin-based motility stage of *L*. *monocytogenes*

Experiments were then performed to investigate whether LisCVs are formed in the first infected cells, such as SLAPs in macrophages, or in cells that are secondarily infected following cell-to-cell spread. JEG3 cells were preferentially used to address this question as these cells grow in a monolayer, which is suitable to observe cell-to-cell spread with microscopy. The effects of different strains and MOIs on the viability of JEG3 cells was first examined. As shown in S3A Fig, the best condition to limit cytotoxicity over time was to infect monolayers of JEG3 cells with strain 10403S using a very low MOI (0.1). At the onset of infection (2h p.i.), the mean number of intracellular bacteria per cell was 0.05±0.01. This low internalization rate allowed monitoring bacterial dissemination from isolated infected cells to neighboring uninfected cells. After 2h of infection, only a few cells were infected by at most one intracellular bacterium, as shown with a double-immunofluorescence staining that distinguishes between extracellular and intracellular bacteria (S3B Fig). Low-magnification microscopy highlighted the dissemination of bacteria from the initially infected cell (2h p.i.) to adjacent cells forming isolated infection foci (6h p.i.), which assembled thereafter (24h p.i.), consistent with the active multiplication and spreading of bacteria (S3C Fig). At 72h p.i., most cells were infected and the integrity of the cellular monolayer was largely preserved, as revealed by DAPI and actin staining. Only few lytic plaques were observed, although 20–30% of nuclei looked damaged (S3D Fig). In the meantime, there was a 50-fold reduction in CFU between 24h and 72h p.i. (S3E Fig). High-magnification microscopy and quantification of actin–and LAMP1–associated bacteria confirmed that bacteria switched from actin-based motility at 6h p.i. (66±4% of Actin^+^ LAMP1^-^ bacteria) to LisCVs at 72h p.i. (79±2% of Actin^-^ LAMP1^+^ bacteria) (S3F Fig).

In order to get a dynamic view of the process, infection of JEG3 cells by fluorescent mCherry-expressing 10403S bacteria was monitored using live cell imaging. The results confirmed that bacteria disseminated from the initially infected cells to the entire cell monolayer during the first day of infection. Thereafter, bacteria became progressively confined to perinuclear regions (S1 Movie). Highly infected cells rounded up and detached from the coverslip. In contrast, secondarily infected cells spread on the coverslip and harbored perinuclear bacteria, as observed in fixed cells (three representative cells are indicated with arrows in S1 Movie, from 28h to 73h p.i.). These results suggested that LisCVs are not formed in the first infected cells and, consequently, do not originate from internalization vacuoles.

**Fig 2:**
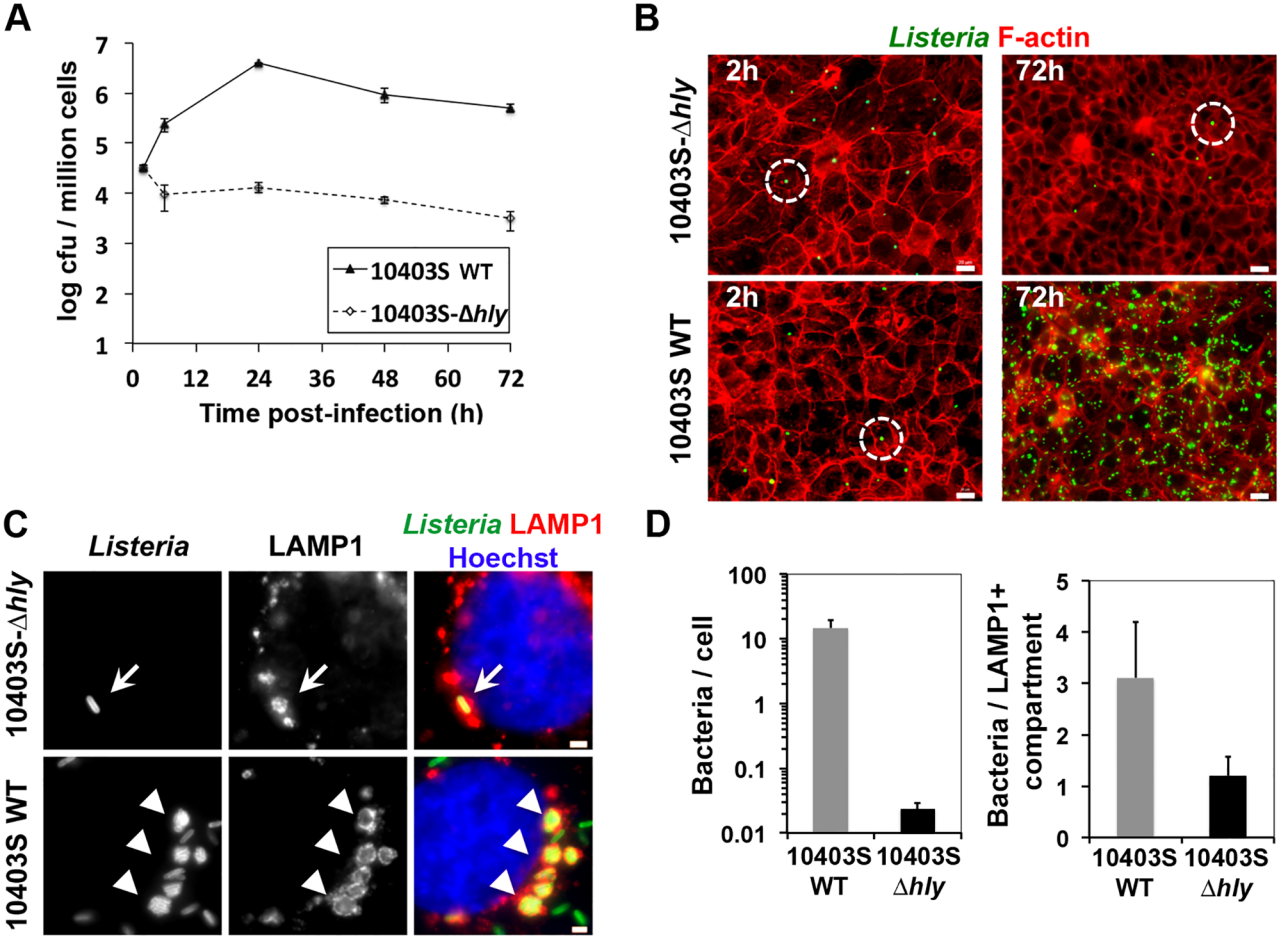
LLO-deficient bacteria remain confined in internalization vacuoles. JEG3 cells were infected with *L*. *monocytogenes* 10403S wild type (WT) or 10403S-Δ*hly* bacteria (MOI ~ 0.1) and lysed to determine bacterial intracellular loads by CFU counts, or processed for microscopy at the indicated time. **A**. Bacterial growth curves. Results are mean±SD of triplicate experiments. **B**. Low magnification micrographs of JEG3 cells infected for 2h or 72h. Images are overlays of *Listeria* (green) and F-actin (red) signals. Circles highlight a single bacterium within a host cell. Images have been digitally processed to enhance the green fluorescent signal. Bars: 20 μm. **C**. High magnification micrographs of infected cells at 72h p.i. show a representative LAMP1^+^ compartment encircling a single Δ*hly* bacterium (arrow), or several LisCVs encircling several WT bacteria (triangles). Overlays show *Listeria* (green), LAMP1 (red) and Hoechst (blue) signals. Bars: 2 μm. **D**. Histograms of the number of intracellular bacteria per cell (left) or per LAMP1^+^ compartment (right). At least 1000 cells were examined per experiment. Results represent mean±SD of triplicate experiments.

To confirm that LisCVs are not internalization vacuoles, we also studied the fate of a *hly* mutant strain, which does not produce the cytolysin LLO and fails to escape the entry vacuole in most cell types [36]. The strain 10403S-Δ*hly* entered JEG3 cells as efficiently as wild type (WT) bacteria (Fig 2A), but the intracellular load of 10403S-Δ*hly* bacteria declined after 6h and was about 100-fold less than that of wild-type bacteria at 72h p.i. Low-magnification microscopy confirmed that the 10403S-Δ*hly* mutant did not disseminate in the cell monolayer (Fig 2B). The few Δ*hly* bacteria remaining after 3 days of infection were isolated bacteria enclosed in LAMP1^+^ compartments (Fig 2C and 2D). In contrast, WT bacteria that had spread in the cell monolayer (Fig 2B) were enclosed in several LisCVs (6 ± 2 LisCVs per cell), each LisCV containing several bacteria (mean of 3 bacteria per LisCV, Fig 2C and 2D). These results indicate that LLO-deficient bacteria remain confined in entry vacuoles, which fuse with LAMP1^+^ compartments as in macrophages [37], but do not form LisCVs.

To demonstrate that bacteria present in LisCVs have entered the cytosol at some point during the infection process, we employed the CBD-YFP probe that specifically detects cytosolic *L*. *monocytogenes*, as described previously [37–39]. At 6h pi, CBD-YFP-labeled bacteria were efficiently detected in transfected cells (S4A Fig), as well as in surrounding non-transfected cells, indicating that the probe remained at the surface of motile bacteria during cell-to-cell spread. Accordingly, infection foci also contained CBD-YFP-labeled bacteria after 24h of infection. At 72h p.i., the CBD-YFP signal associated with intracellular bacteria decreased, but CBD-YFP dots remained visible on the surface of bacteria found within LAMP1^+^ compartments, particularly at bacterial poles (S4A and S4B Fig). Taken together, these results show that *L*. *monocytogenes* escapes into the host cytosol and spreads from cell to cell before being engulfed in vacuoles.

### The formation of LisCVs coincides with the disappearance of ActA from the bacterial surface

**Fig 3:**
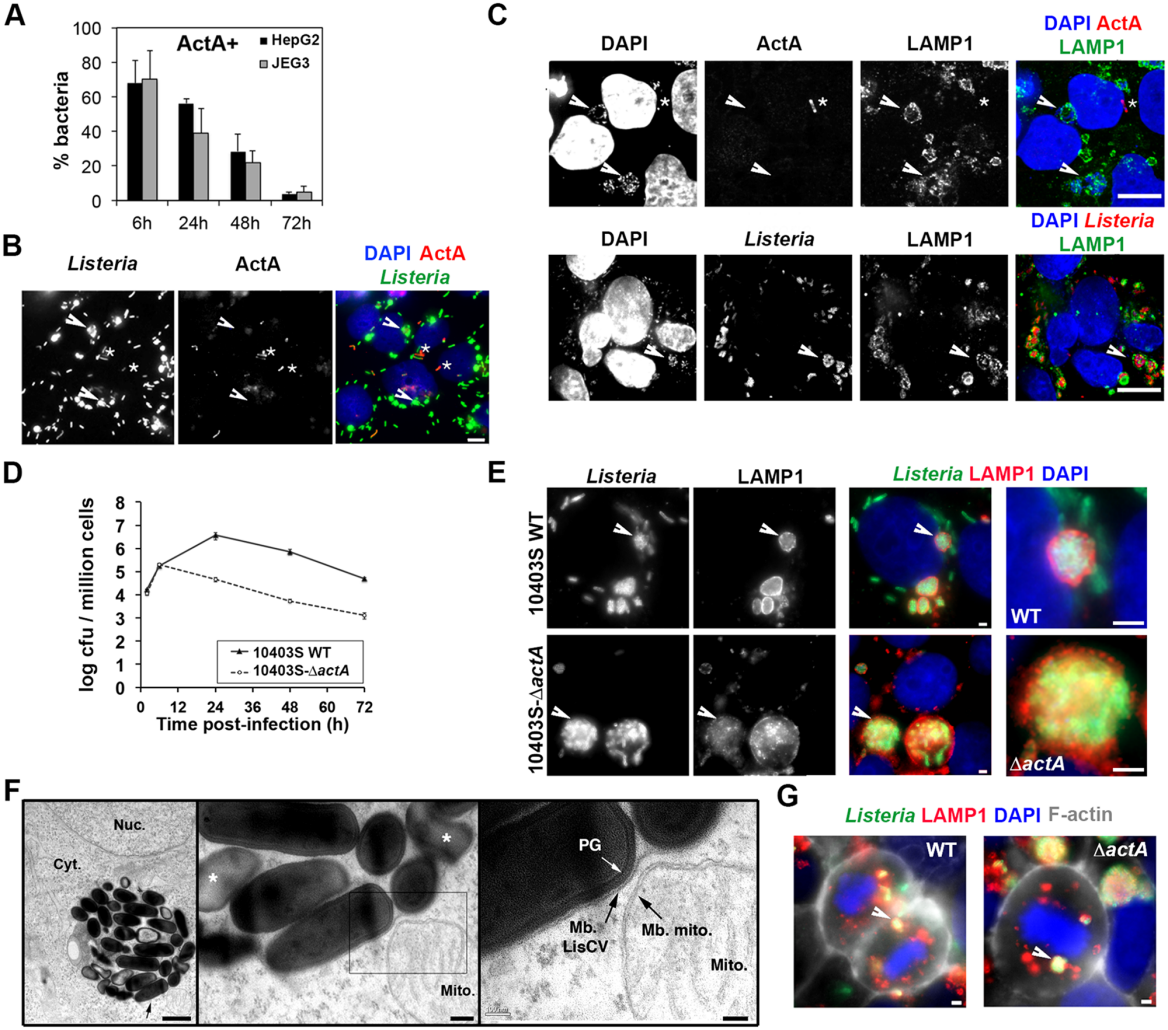
The formation of LisCVs is associated with ActA deficiency. **A-C** Cells were infected with *Listeria* EGDe for 72h (HepG2, MOI ~ 1; JEG3 MOI ~ 0.1) and labeled with *Listeria* polyclonal and ActA monoclonal antibodies. **A**. Histograms of the percentage of ActA–positive bacteria. Data are mean±SD of triplicate experiments. At least 500 bacteria were observed per time-point. **B-C**. Micrographs show representative cells labeled with antibodies against *Listeria*, ActA and/or LAMP1 and DAPI to visualize bacterial nucleoid and cell nuclei. For the overlay images, the color of each staining is indicated on the panel headlines. Arrows point groups of ActA-negative bacteria in LisCVs. Stars (*) indicate examples of ActA–positive bacteria. Bars: 10 μm. **D-E**. JEG3 cells monolayers were infected for 72h with *L*. *monocytogenes* 10403S wild type (WT) or 104033S-Δ*actA* strain (MOI ~ 0.1) in triplicate experiments. **D**. Intracellular growth of bacteria assessed by CFU counts. **E**. Representative LisCVs in cells infected with WT or Δ*actA* bacteria. Arrows point LisCVs showed at a higher magnification on the right. Bars: 2 μm. **F**. TEM micrographs show EGDe-Δ*actA* bacteria in a vacuole at three magnifications. Bars: 1 μm; 0.2 μm; 0.1 μm. Black arrows point the single membrane of the vacuole (Mb. LisCV) and the double-membrane of a neighboring mitochondrion (Mb. mito.) The white arrow points the peptidoglycan (PG) of an intact *Listeria*. Altered bacteria are indicated by *. Nuc., nucleus; Cyt., cytosol. **G**. LAMP1^+^ bacteria in mitotic cells. Micrographs are overlays of *Listeria* (green), LAMP1 (red), F-actin (white) and DAPI (blue) signals and are representative of mitotic cells observed in 10 independent experiments. Bars: 2 μm. White arrows point to LAMP1^+^ *Listeria*.

The expression of the gene encoding the actin-nucleating factor ActA is significantly induced when bacteria escape from the primary vacuole and enter into the host cytosol [40], but the fate of ActA during a prolonged infection is unknown. Since bacteria trapped in LisCVs were not associated with F-actin, we hypothesized that these bacteria no longer express ActA. Immunostaining for ActA revealed that the percentage of ActA-positive (ActA^+^) bacteria decreased from about 70% to 5% in both HepG2 and JEG3 cells between 6h and 72h p.i. (Fig 3A and 3B). At 72h p.i., no LAMP1^+^ bacteria were labeled with ActA antibodies (Fig 3C). The decrease in the number of ActA^+^ bacteria over time matched the decline in Actin^+^ bacteria and began at 24h p.i., before the drastic increase in LAMP1^+^ bacteria observed between 48h and 72h p.i. (Fig 1C). These results suggest that bacteria enter the cytosol and non-synchronously cease producing ActA, before being incorporated in LAMP1^+^ compartments.

### Δ*actA* mutant bacteria also occupy vacuoles after long-term infection

The transition of *L*. *monocytogenes* from the host cytosol to LAMP1^+^ compartments correlated with a decrease in ActA expression during long-term infection. However, it was possible that LisCVs derived from secondary vacuoles following cell-to-cell spread. To test this hypothesis, long-term infections were performed with 10403S-Δ*actA*, a strain defective in actin-based motility and cell-to-cell spread. CFU counts indicated that *actA*-deficient bacteria replicated for the first 6 hours of infection. Then the number of intracellular bacteria decreased in the same proportion (40-fold) as the WT bacteria at 72h p.i. (Fig 3D). Surprisingly, observation of bacteria by microscopy did not show any decrease in the number of Δ*actA* bacteria labeled with *Listeria* antibodies. Moreover, though non-motile, Δ*actA* bacteria were not constrained to the initially infected cells and spread to a few neighboring cells (S5 Fig), forming small infectious foci at 72h p.i. These foci showed bacteria trapped in LAMP1^+^ compartments, which were often larger and contained more bacteria than those generated during infection by the WT strain (Fig 3E). Electron microscopy confirmed that Δ*actA* bacteria were present within single-membrane vacuoles, enclosing mixed populations of intact and damaged bacteria (Fig 3F). A possible cause of the slight intercellular dissemination of Δ*actA* bacteria could be a transport of vacuoles during the division of host cells, as suggested by the detection of LAMP1^+^ WT and Δ*actA* bacteria in some mitotic cells (Fig 3G). Together, these results indicate that, during a prolonged infection, *actA*-deficient bacteria become trapped in LisCVs, which may be transferred from cell-to-cell during mitosis.

### LisCVs have characteristics of lysosome-like organelles

The sequestration of cytosolic *Listeria* by host endomembranes resembled an autophagic process. This hypothesis was supported by the fact that Δ*actA* mutants are targeted by autophagy-related processes in MDCK cells [14] and macrophages [15]. In addition, Δ*actA* mutant have been shown to replicate as efficiently as their parent strains within the first day of infection in macrophages, suggesting that, if taken by the autophagy pathway, they avoid destruction in autolysosomes [41]. Since the link between *Listeria* and the autophagy machinery has not yet been studied in human epithelial cells, we examined the colocalisation of the autophagy marker LC3 with 10403S or EGDe. Strain 10403S displayed negligible recruitment of LC3 throughout the three-day infection in JEG3 cells (S6A and S6B Fig). Similarly, at 72h p.i., EGDe bacteria did not recruit LC3 in HepG2 cells (99±0.5% of bacteria were LC3-negative; n = 3). In JEG3 cells expressing GFP-LC3, a fusion protein that marks autophagic membranes [14, 15, 38], there was no noticeable overlap between GFP-LC3 and LAMP1^+^ EGDe bacteria (S6C Fig). The role of autophagy in the formation of LisCVs was also tested by knocking down BECLIN1 or ATG7, which are involved in the nucleation and elongation steps of autophagosome formation, respectively [42]. Transfection with BECLIN1 or ATG7 siRNA was efficient in reducing the expression levels of *BECN1* and *ATG7* transcripts (S6D Fig), as previously described [43], but did not reduce the number of LAMP1^+^ bacteria (S6E Fig) and did not impact on CFU counts at 72h p.i. (S6F Fig). Overall, these results suggest that canonical autophagy is not involved in the formation of LisCVs, but do not exclude a role for a non-canonical pathway.

**Fig 4:**
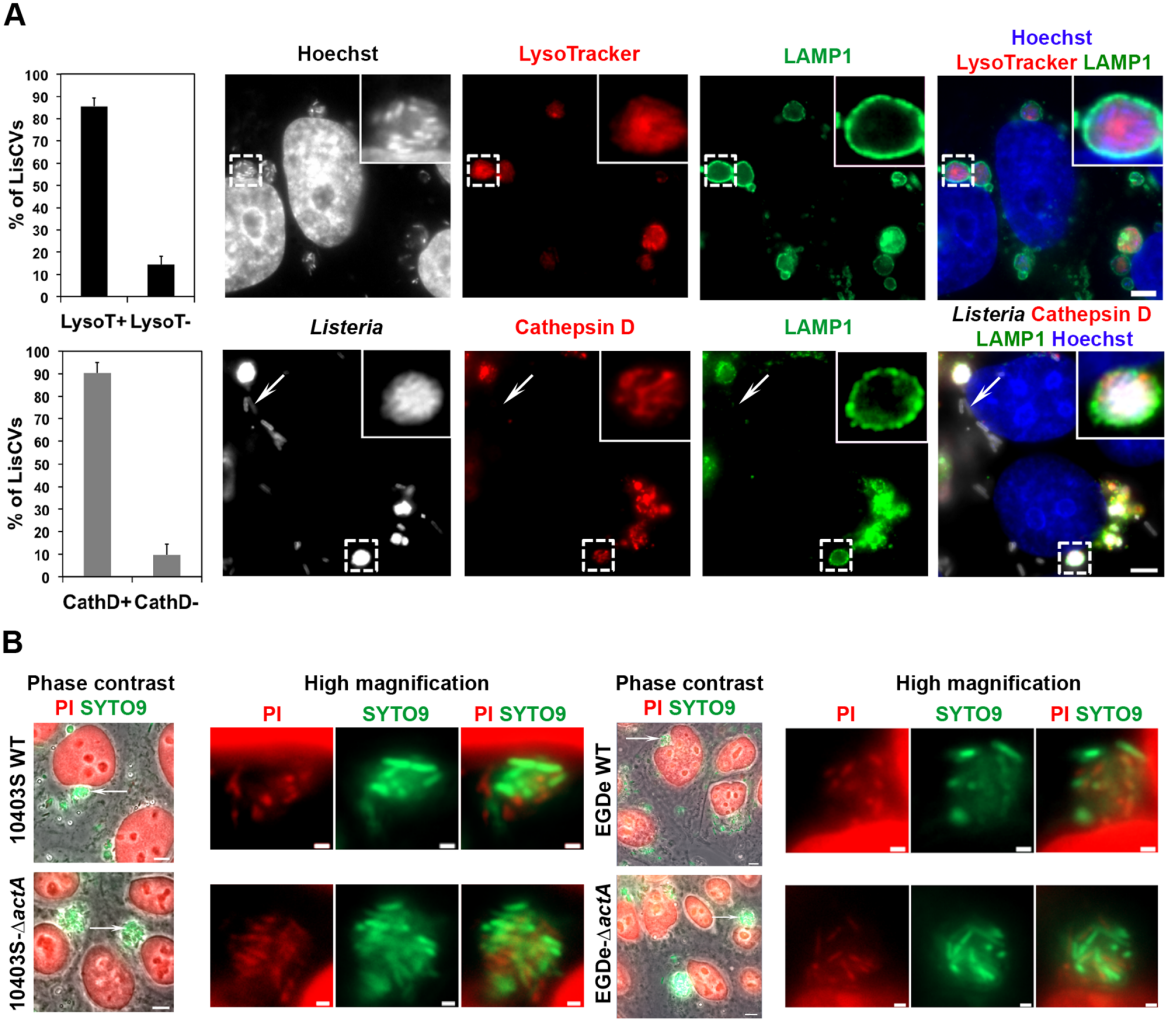
LisCVs have lysosomal features. JEG3 cells monolayers were infected for 72h with the indicated *L*. *monocytogenes* strain (MOI ~ 0.1). **A**. Cells infected with 10403S were fixed and labeled with Hoechst or *Listeria* antibody (to detect bacteria), LysoTracker or cathepsin D antibody, and a LAMP1 antibody. Histograms represent the % of LisCVs positive or negative for the indicated marker. Results are mean±SD of triplicate experiments. Representative micrographs are shown beside. The color of each staining is indicated on the panel headlines (bars: 5 μm). A framed LisCV is shown at a higher magnification in the upper right corner. The arrow points a cathepsin D-negative bacterium. **B**. Cells were infected with the indicated WT or Δ*actA* strains, permeabilized with 0.1% Triton X-100, double-labeled with SYTO9 and Propidium Iodide (PI) and examined under the microscope. Phase contrast shows groups of cells. Bacteria with intact membranes are stained in green, while the host cell nuclear DNA and damaged bacteria are stained in red. Bar: 5 μm. High-magnifications of regions pointed by arrows are shown on the right. Images are representative of 3 independent experiments. Bar: 1 μm.

LisCVs are bound by a single membrane and contain electron-dense heterogeneous materials and membranes structures (Fig 1F and S2 Fig), suggesting that these compartments have fused with lysosomes. In addition, TEM studies highlighted some bacteria that may be in the process of being captured by electron-dense organelles resembling secondary lysosomes (S2 Fig, lane 3). To further characterize these vacuoles, we used LysoTracker, a dye that marks acidic organelles including lysosomes [44], and an antibody that detects the lysosomal protease cathepsin D. In fixed JEG3 cells infected for 72h with 10403S, 85% and 89% of LisCVs were stained with LysoTracker and the cathepsin D antibody, respectively (Fig 4A). These results were confirmed in live JEG3 cells infected with either GFP-expressing WT or Δ*actA* bacteria (S7A Fig), and in fixed JEG3 cells infected with Δ*actA* bacteria (S7B Fig). Thus, LisCVs have lysosomal features.

Since lysosomes are degradative organelles, we also asked whether *L*. *monocytogenes* could survive in LisCVs. As previously noticed, the bacterial GFP fluorescence was preserved in many vacuolar bacteria, indicating that these bacteria were not were not lysed (Fig 1H; S7A Fig). At the same time, quantitative TEM indicated that 26% of vacuolar bacteria were degraded (Fig 1G). The viability of intravacuolar bacteria was assessed with the LIVE/DEAD BacLight assay. This assay is based on the discriminative labeling of two fluorescent nucleic acid-stains: the green membrane-permeant dye SYTO9 and the red membrane-impermeant dye propidium iodide (PI), which only crosses damaged cell membranes. Both dyes penetrate dead cells, but SYTO9 fluorescence is reduced in presence of PI. As such, live bacteria appear green, while those with damaged membranes appear red [45, 46]. To allow the dyes to reach vacuolar *Listeria*, this staining protocol was performed in the presence of 0.1% Triton X100, which permeabilizes host cell membranes, including vacuoles containing bacteria [47]. The efficiency of permeabilization was demonstrated by the bright staining of host cell nuclei with PI, as the dye crossed the double-membrane of the nuclear envelope. Both wild type and Δ*actA* strains formed clusters of green and red bacteria in perinuclear regions, indicating that LisCVs contain mixed populations of bacteria with intact and damaged membranes (Fig 4B), in agreement with quantitative TEM data (Fig 1G). These results reveal that a significant portion of bacteria that reside in LisCVs resist the degradative function of these lysosome-like organelles.

### *L*. *monocytogenes* can return to an active state of replication and motility following subculture of host cells

**Fig 5:**
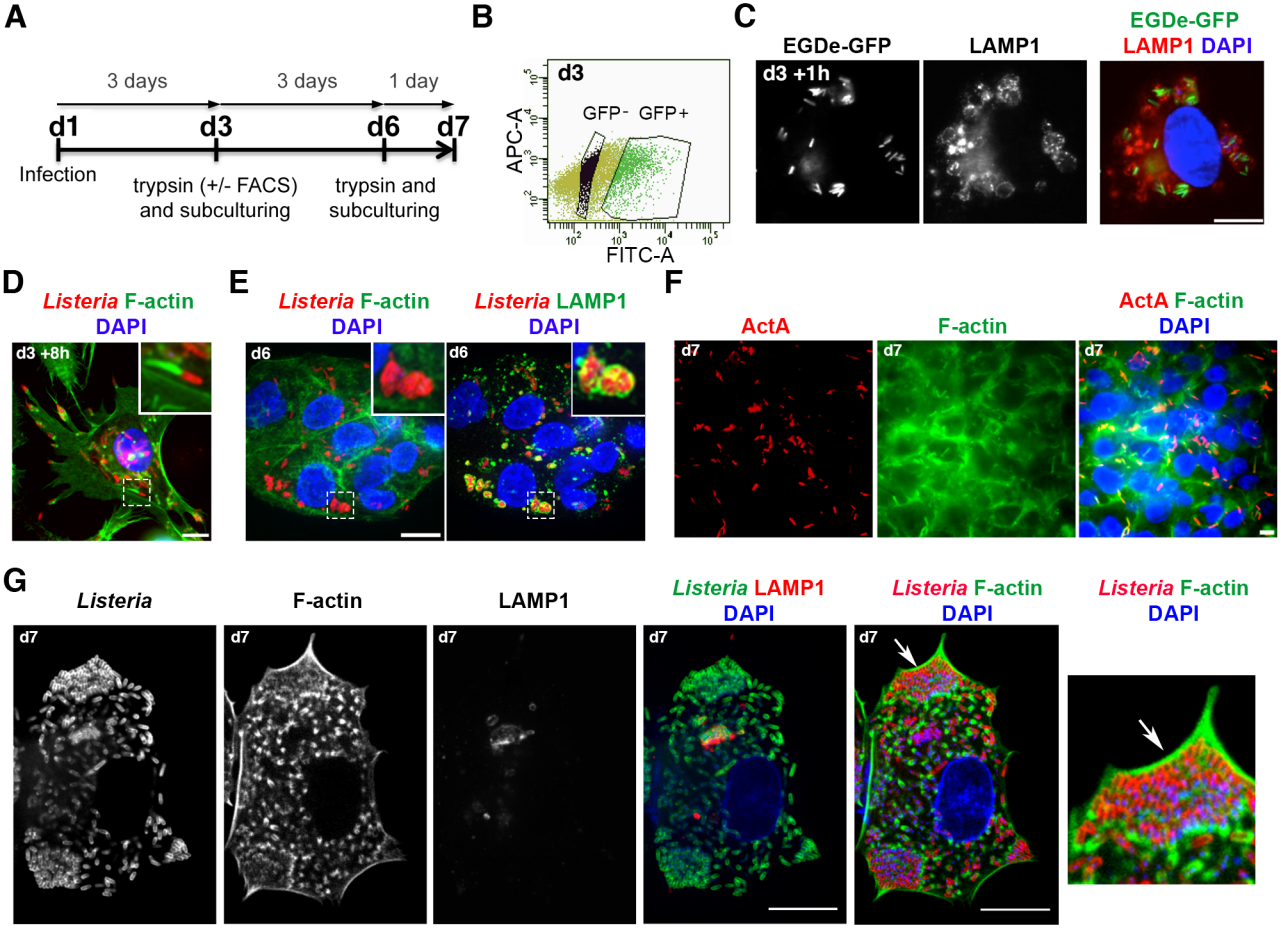
*L*. *monocytogenes* cycles from vacuolar to cytosolic stages during cell subculturing. **A**. Experimental design of cell subculturing. (“d”: day). **B-C**. HepG2 cells containing EGDe-GFP bacteria entrapped in LisCVs were purified by FACS (B), plated and examined by microscopy 1h later (d3+1h, C). (C) GFP-positive bacteria (green) are present in LAMP1^+^ compartments (red) near nuclei (blue). Bar: 10 μm. **D-E**. The same cells were examined 8h (d3+8h) and 3 days later (d6). Micrographs show representative images of cells stained with *Listeria* antibodies (red), fluorescent phalloidin to label F-actin or LAMP1 antibodies (green) and DAPI (blue). Bar: 10 μm. The framed regions are shown at a higher magnification in the upper right corner. **F**. The same cells were examined after another cell passage and 1 day of growth (d7) and labeled with ActA antibodies, fluorescent phalloidin and DAPI. Bar: 10 μm. **G**. JEG3 cells were infected with *Listeria* EGDe and grown as in (A) up to d7. The overlay images show confocal micrographs of *Listeria* or F-actin (green), *Listeria* or LAMP1 (red) and DAPI (blue). Bacteria heavily replicated in the cytosol, were concentrated at the edge of the host cell and were associated with short actin tails. Bar: 10 μm. A magnified image of the region pointed by an arrow is shown on the right.

We then sought to determine whether *L*. *monocytogenes* has the ability to survive in LisCVs for a longer period of time. Considering that human cells need to be subcultured regularly in order to remain alive for a long time, a fluorescence-activated cell sorting (FACS)-based protocol was developed to enrich and subculture cells hosting EGDe-GFP in LisCVs (Fig 5A and 5B). Sorted HepG2 infected cells were plated in complete medium containing 25 μg/mL gentamicin, which prevents reinfection by extracellular bacteria. One hour after passaging, all sorted cells mainly contained LAMP1^+^ bacteria (85±5%; n = 3) (Fig 5C). After 8h of growth, cells were surprisingly divided into two distinct populations: those that still contained a majority of LAMP1^+^ bacteria, and those that contained a majority (over 50%) of actin-polymerizing bacteria (Fig 5D). Since bacterial degradation was not observed in the cytosol of these cells, it was concluded that bacteria had escaped LisCVs and re-entered the cytosolic stage of their intracellular lifecycle. These cells were subsequently monitored for three days (d6), during which only remained a population of cells containing a majority of LAMP1^+^ bacteria (80±3%, n = 3) (Fig 5E). After another cell passage and 24h of growth (d7), there were again two cell populations, one carrying vacuolar bacteria and the other harboring more than 50% of bacteria polymerizing actin (S8 Fig), which were ActA-positive (Fig 5F). By reproducing this experiment five times, we found that, intriguingly, the proportion of each cell population varied widely from one experiment to another (from 10% to 50%). These results showed that vacuolar bacteria could survive after more than three days of infection, but also had the ability to exit vacuoles and return to the active motility phase. Similar results were obtained in JEG3 cells infected with EGDe and subcultured following detachment with trypsin (without the FACS procedure). At day 7, from 20% to 80% of cells contained large amounts of actin-associated bacteria. Confocal microscopy highlighted bacteria with small actin tails mostly concentrated at the leading edge of the cell (Fig 5G). These results reveal that subculture of infected cells promotes a heterogeneous cycling of *L*. *monocytogenes* between vacuolar and cytosolic phases.

### ActA-deficient bacteria persist in host cells as VBNC forms

**Fig 6:**
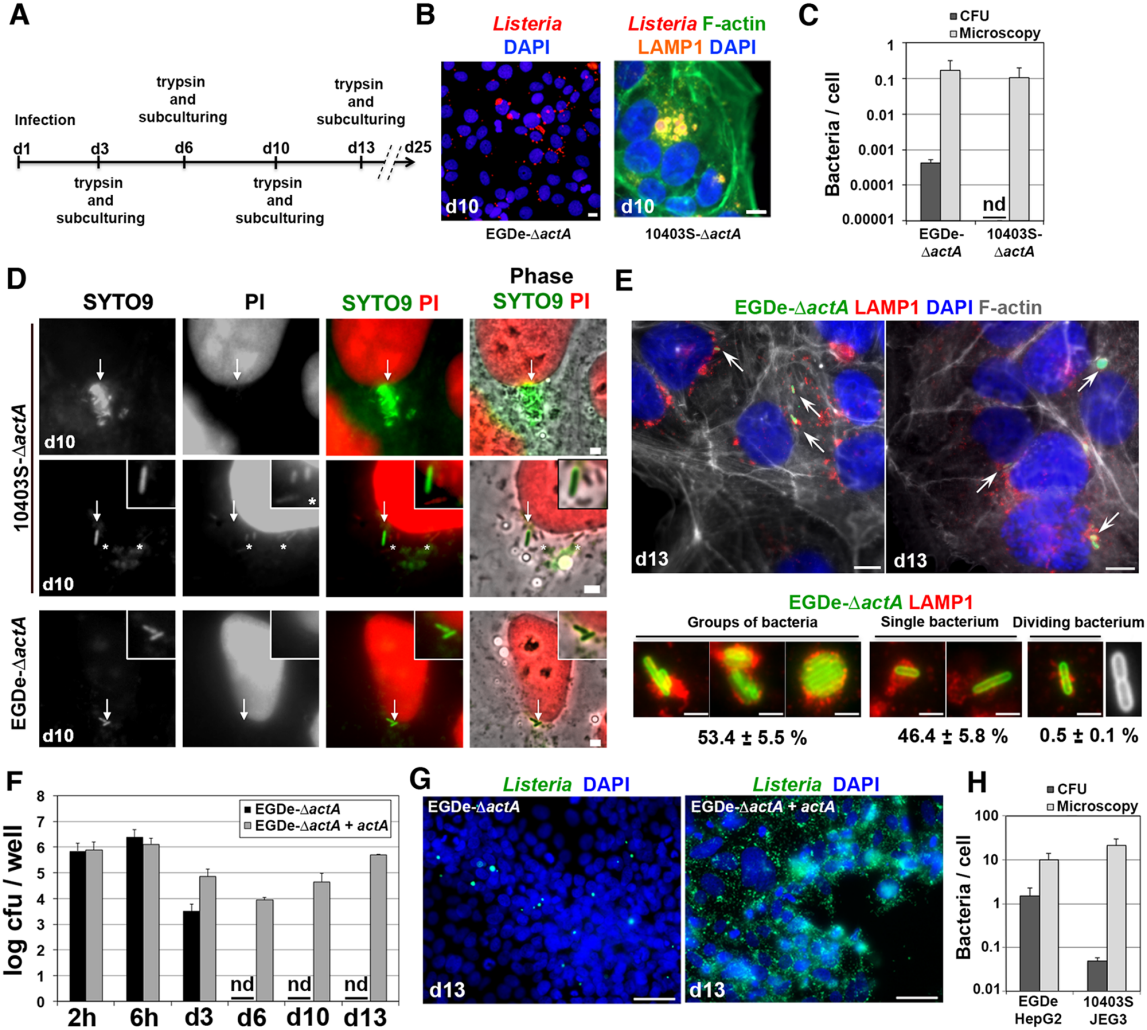
ActA deficiency promotes intracellular persistence of *Listeria* in a VBNC state. **A**. JEG3 cells were infected with Δ*actA* strains (MOI ~ 1) for 3 days (d3) then passed and propagated as indicated. **B**. Representative micrographs of EGDe-Δ*actA* or 10403S-Δ*actA* subcultured for 10 days (d10), with two cell passages (at d3 and d6). The color of each staining is indicated on the panel headlines. Bars: 10 μm. **C**. Comparison of CFU- and microscopic-count methods for the quantification of intracellular Δ*actA* bacteria at d10. Data are mean±SD from three wells in two independent experiments. 10403S-Δ*actA* did not form any colony (nd: not detectable). **D**. Infected JEG3 cells at d10 were permeabilized with 0.1% Triton X-100 and double-labeled with SYTO9 and PI. Intact *Listeria* cells are stained in green (arrows), while damaged bacteria (*) and nuclei are stained in red. Bar: 1 μm. Squared boxes show higher magnifications. Images are representative of 3 independent experiments. **E**. Micrographs of JEG3 cells harboring EGDe-Δ*actA* at day 13. Two representative fields of independent experiments are shown. The color of each staining is indicated on the panel headlines. Bars: 10 μm. Bacteria pointed by arrows are shown at a higher magnification below. A dividing bacterium is highlighted in white. Bars: 2 μm. The % of each bacterial category is indicated as mean±SD of triplicate experiments. **F**. JEG3 cells were infected with EGDe-Δ*actA* or EGDe-Δ*actA+actA* at MOI ~ 1. Infected cells were propagated for 13 days with cell passages at d3, d6 and d10 and cell lysates were plated before each passage and at d13. Data are mean ± SD of triplicate experiments. EGDe-Δ*actA* is in a VBNC state from d6 to d13. nd: not detectable. **G**. Representative micrographs of JEG3 cells infected with EGDe-Δ*actA* or EGDe-Δ*actA+actA* at d13, after staining with DAPI (blue) and *Listeria* antibodies (green). **H**. Quantification of wild type intracellular *L*. *monocytogenes* by CFU or immunofluorescence-labeling of bacteria. HepG2 cells were infected with strain EGDe (same experiment as in Fig 1A) and JEG3 cells were infected with strain 10403S (same experiment as in S3C Fig). Intracellular bacteria were quantified by CFU counts or microscopy. Data are mean ± SD of triplicate experiments.

In contrast to wild type bacteria, Δ*actA* bacteria cannot switch back to actin-based motility during cell subculturing. By studying the behavior of Δ*actA* mutants within cells propagated via several passages, one may study the long-term fate of vacuolar *L*. *monocytogenes* in the long term. JEG3 cells infected with EGDe-Δ*actA* or 10403S-Δ*actA* bacteria were grown for three cycles of three to four days (Fig 6A). On day ten, many cells remained infected by Δ*actA* bacilli associated with LAMP1 (Fig 6B). Some of these vacuolar bacteria were observed in dividing cells at different stages of mitosis (S9 Fig), as previously observed after three days of infection (Fig 3G). These results suggested that bacteria in vacuoles might be transmitted upon host cell division.

We quantified the number of vacuolar Δ*actA* bacteria at day 10 by plating cell lysates on BHI agar plates. This generated very few (EGDe-Δ*actA*) or no (10403S-Δ*actA*) bacterial colonies, even after one week of incubation at 37°C. However, at the same time intact intracellular bacilli were observed by microscopy. In comparison to the quantification of bacteria using microscopy, CFU counts severely underestimated the number of intracellular bacteria (Fig 6C). These results suggested that *actA*-deficient bacteria were still viable but unable to grow on BHI plates. The inability of bacteria to grow on standard culture media, although they are still viable and maintain a low metabolic activity, defines the VBNC state [48, 49]. The viability of intracellular Δ*actA* bacteria was assessed with the use of the LIVE/DEAD BacLight assay, which detect VBNC bacteria [50]. At day 10, most intracellular Δ*actA* bacteria stained green, indicated that these bacteria were not damaged (Fig 6D). Yet, bacteria present in cell lysates from parallel experiments did not grow on BHI agar plates or in BHI liquid medium.

Assuming that if these bacteria were alive, they should not be eliminated after another cell passage, infected cells were further propagated for a total of 13 days of infection (4 cycles of subculturing, Fig 6A). LAMP1^+^ bacteria were still observed at this time-point, but no colonies were detected on BHI agar plates. Quantification of intracellular bacteria by immunofluorescence staining indicated a mean of 0.051 ± 0.007 bacteria per cell (~ 2000 cells were analyzed in each independent experiment; n = 3) for a total of about 50 000 bacteria per well. Of these bacteria, 53% were found in bacterial clumps, 46% were isolated and 0.5% showed a division septum (Fig 6E). This suggested that Δ*actA* bacteria found in cells after 13 days of infection had a low metabolic activity and divided slowly. Infected cells were subsequently propagated for many generations in order to determine whether VBNC bacteria are ultimately eliminated along cell passages. Rod-shaped *L*. *monocytogenes* bacteria were still detected in host cells after 25 days of subculture (seven passages), but no colonies were detected on BHI agar plates. These results indicate that Δ*actA L*. *monocytogenes* persisted in host cells in a VBNC state.

We assessed whether this phenotype was reversible upon re-expression of the *actA* gene in the EGDe-Δ*actA* mutant, by site-specific chromosomal integration of *actA* in the tRNA^Arg^ locus [51, 52]. We noticed that in this strain (EGDe-Δ*actA+actA*), *actA* was not regulated as in the wild type EGDe strain, since after 3 days of infection in JEG3 cells, most EGDe-Δ*actA+actA* bacteria produced ActA at the bacterial surface and polymerized actin, in contrast to the wild type strain (S10 Fig). This discrepancy in ActA expression between EGDe and the complemented strain might be explained by the complex regulation of *actA* expression [51]. This strain that produced sustained levels of ActA was used to assess the importance of ActA repression for the acquisition of an intracellular VBNC state by *L*. *monocytogenes*. In contrast to EGDe-Δ*actA* bacteria, EGDe-Δ*actA+actA* bacteria were still culturable on BHI plates after 13 days of cell propagation (Fig 6F) and continued replicating, polymerizing actin and spreading from cell-to-cell leading to many infected cell foci and lytic plaques (Fig 6G). Together, these results demonstrate that the repression of ActA is required for the acquisition and maintenance of the VBNC state during long-term infection of host cells by *L*. *monocytogenes*.

### Evidence for the emergence of VBNC bacteria during prolonged infection of epithelial cells with wild-type *L*. *monocytogenes*

Similarly to Δ*actA* mutants, we speculated that wild-type strains of *L*. *monocytogenes* also enter a VBNC state during long-term intracellular infection. Since cellular subculturing induced the reactivation of wild-type *L*. *monocytogenes*, propagation of cells was avoided and the number of intracellular bacteria was estimated after a three-day infection, both by CFU counts and by microscopy. The number of EGDe bacteria found in HepG2 cells as evaluated by CFUs was 7-fold lower than that determined by microscopy. The discrepancy between these two quantification methods was even larger during the infection of JEG3 cells by 10403S (i.e. on the order of 2 logs) (Fig 6H). These data support the hypothesis that LisCVs contain bacteria in different physiological states, including bacteria that grew on plates, bacteria that have reached a VBNC state and dead bacteria.

### Gentamicin selects persistent forms of *L*. *monocytogenes*

**Fig 7:**
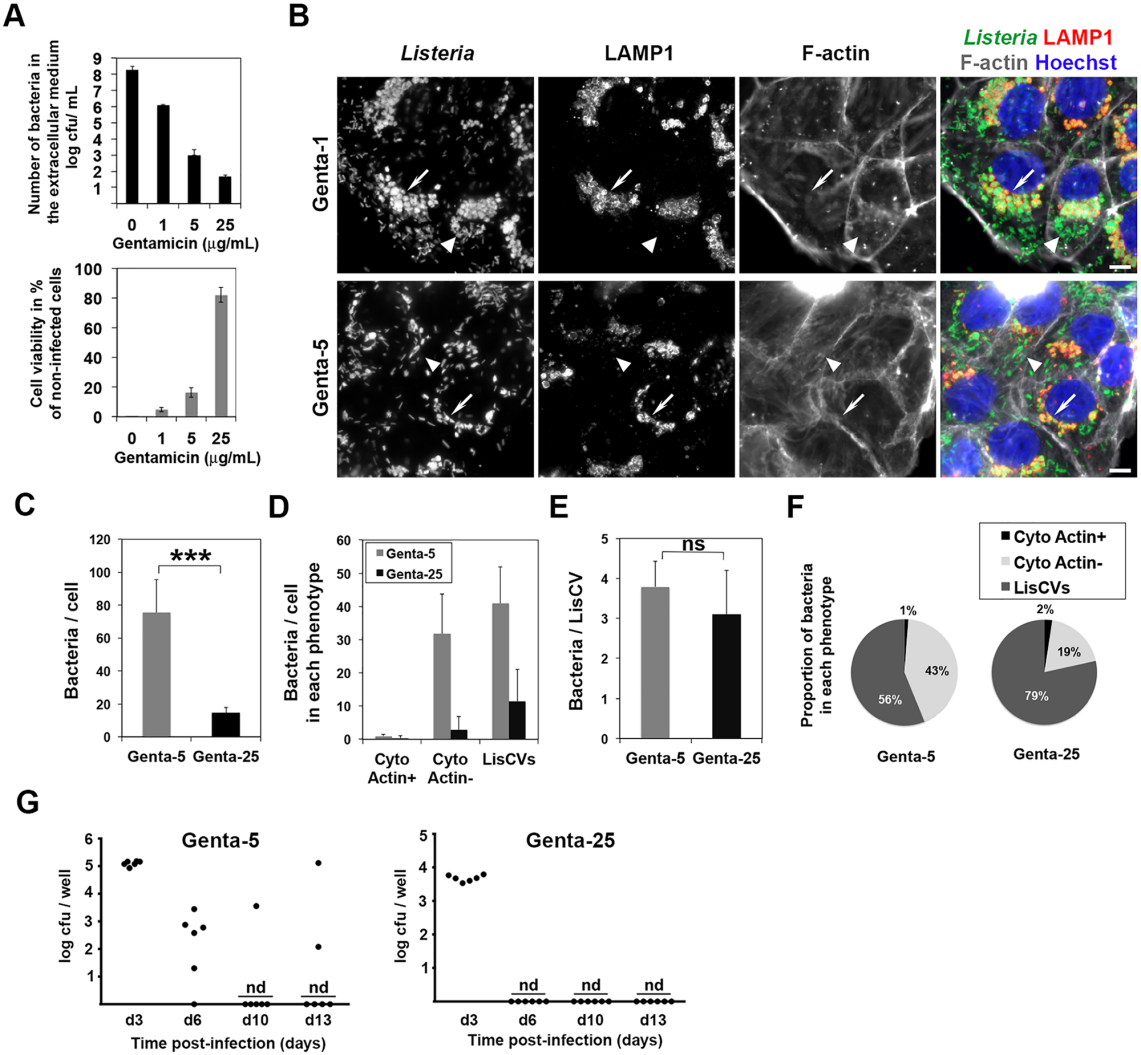
A high concentration of gentamicin favors the selection of *L*. *monocytogenes* persistent forms. **A-F**. JEG3 cells were infected with *L*. *monocytogenes* 10403S (MOI ~ 0.1; without 10-min exposure to gentamicin 100 μg/mL), and incubated 72h in presence of different concentrations of gentamicin (0, 1, 5 or 25 μg/mL). Experiments were performed in triplicates. **A**. Number of bacteria in the extracellular medium (by CFU counts) and viability of host cells (represented as a percentage of infected versus uninfected live cells scored by a trypan blue assay). **B**. Representative micrographs of infected cells grown in 1 or 5 μg/mL gentamicin. White arrows show groups of LisCVs; triangles point actin-free cytosolic bacteria. Bar: 10 μm. **C-F**. Effect of the concentration of gentamicin (5 or 25 μg/mL) on the number of bacteria per cell (**C**), the number of bacteria per phenotype (**D**), the number of bacteria per LisCV (**E**) and the proportion of bacteria in different phenotypes (**F**) (*** *p*<0.0005, “ns”, non-significant, Student *t*-test). **G**. Emergence of VBNC bacteria during the subculture of 10403S-Δ*actA*-infected cells grown in 5 or 25 μg/mL gentamicin (as in Fig 6). Infected cells were propagated for 13 days with passages at d3, d6 and d10. Cell lysates were plated before each passage and at d13. Each dot represents the number of bacteria forming colonies (CFU) in the lysate of a well. nd: not detectable. The results are from triplicate experiments (two wells per experiment).

The long-term infection protocol described above made use of a relatively high concentration of gentamicin (i.e. 25 μg/mL, “Genta-25”) in the culture medium, in order to avoid the extracellular survival of bacteria and re-internalization events, as well as to limit the cytoxicity of the infection. However, it was possible that at this concentration, gentamicin accumulated in epithelial cells and reduced the survival of intracellular bacteria, as previously reported in macrophages [53]. This hypothesis was examined in JEG3 cells infected with *L*. *monocytogenes* 10403S without (“Genta-0”) or with lower concentrations of extracellular gentamicin (1 μg/mL, “Genta-1” or 5 μg/mL, “Genta-5”). In addition, we used a low MOI (0.1), which enables to skip the 10-minute pretreatment of cells with gentamicin 100 μg/mL at the onset of infection. As shown in Fig 7A, the lower the antibiotic dose, the more bacteria were present in the extracellular medium and the more the viability of host cells was altered. Infection reduced cell viability by 100% with Genta-0, 95% with Genta-1, 84% with Genta-5, but only 18% with Genta-25 (Fig 7A). At Genta-1 or Genta-5, cells that survived infection contained more bacteria than those grown with Genta-25, but these bacteria were either actin-free in the cytosol or in LisCVs, indicating that the phenotypic switch occurred independently of the concentration of gentamicin (Fig 7B). Similar results were obtained with EGDe (S11A Fig) and the switch was associated with the loss of ActA (S11B Fig).

We performed a quantitative assessment of the effect of decreasing gentamicin concentration from 25 μg/mL to 5 μg/mL on the number and phenotypes of bacteria at 72h pi. There were about 5-fold more bacteria in cells cultured with Genta-5 than with Genta-25 (Fig 7C), indicating that at a high dose, gentamicin penetrated within cells and affected the growth of intracellular bacteria. However, the LisCV phenomenon was not due to the presence of a high concentration of gentamicin since with the low dose (Genta-5), there were 4-fold more LisCVs than with the high dose (Genta-25) (Fig 7D). Interestingly, the number of bacteria per LisCV was not significantly affected by a high concentration of gentamicin (Fig 7E), which suggested that bacteria that resided in these vacuoles were protected from the action of gentamicin. Moreover, the proportion of vacuolar bacteria was higher with Genta-25 (79%) than with Genta-5 (56%), while the proportion of actin-free cytosolic *Listeria* was conversely lower (Fig 7F).

Altogether, these results show that during prolonged infections with *L*. *monocytogenes*, gentamicin controls the growth of cytosolic bacteria but is not responsible for the formation of LisCVs. These results also suggest that gentamicin selects for vacuolar bacteria by inhibiting the growth of bacteria that replicate in the cytosol of host cells.

We hypothesized that a high dose of gentamicin also promotes the selection of VBNC bacteria during the subculture of cells infected with *actA*-deficient bacteria. The subculture experiment described in Fig 6 was therefore reproduced with strain 10403S-Δ*actA*, this time propagating the infected cells in the presence of Genta-5 or Genta-25. As shown in Fig 7G, the VBNC phenotype still occurred despite the decrease in the concentration of gentamicin at 5 μg/mL, but in a less reproducible manner than with 25 μg/mL gentamicin. There was an inter-experimental and inter-well variation in the ability of *actA*-deficient *L*. *monocytogenes* to enter the VBNC state, when host cells were subcultured in presence of Genta-5. At day 13, the VBNC phenotype appeared in 4 out of 6 experiments, whereas in 2 of these experiments, the bacteria did not enter the VBNC state (Fig 7G). Accordingly, JEG3 cells propagated with Genta-5 contained either LAMP1^+^ bacteria that did not form colonies on BHI plates, as in Genta-25 (S12A Fig), or LAMP1-negative bacteria that massively replicated in the cytosol and were culturable when plated on BHI agar plates (S12B Fig). Taken together, these results indicate that a high concentration of gentamicin does not cause, but selects, the vacuolar and VBNC phenotypes, by specifically inhibiting the growth of cytosolic bacteria.

## Discussion

**Fig 8:**
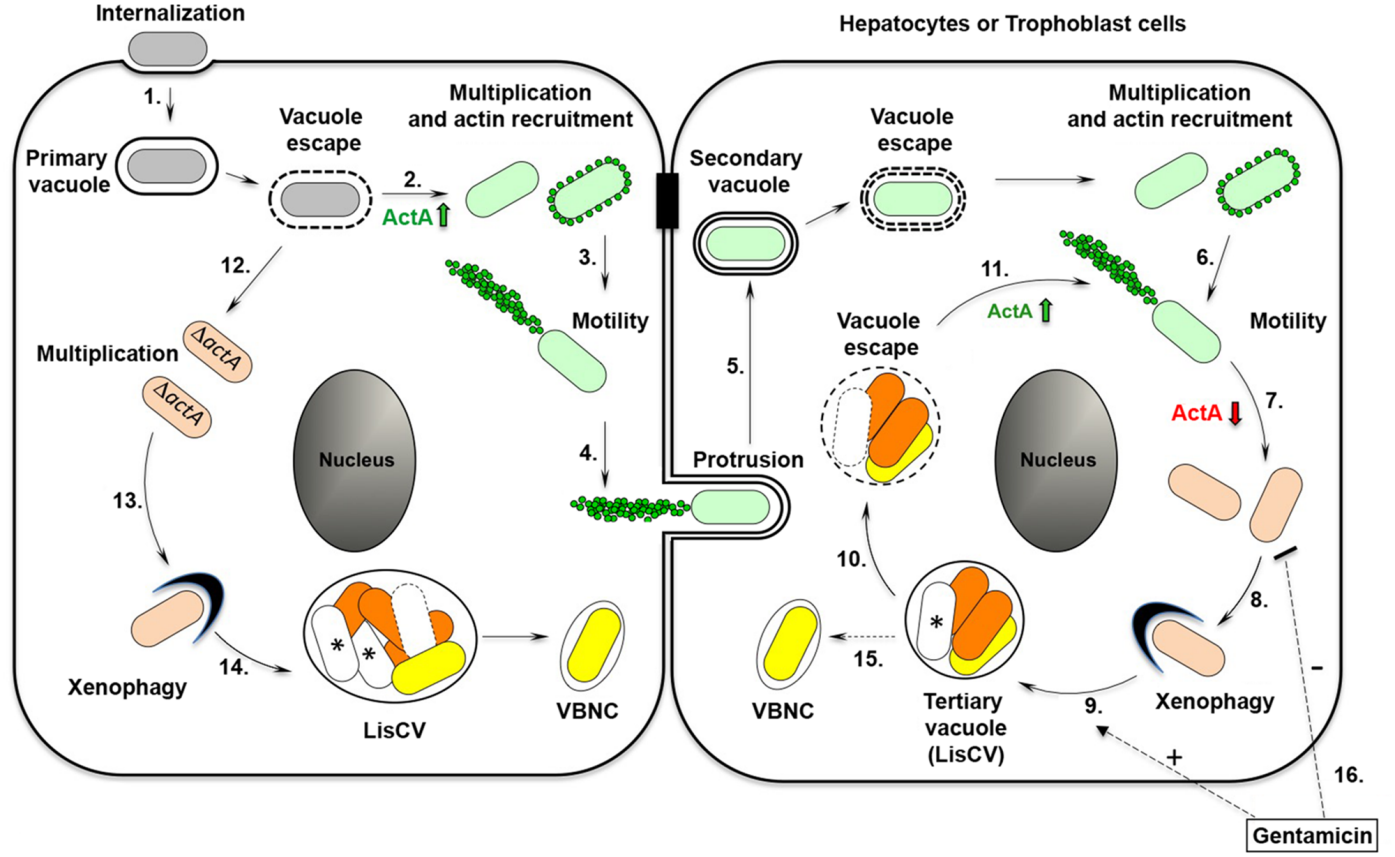
Model for the intracellular life cycle of *L*. *monocytogenes* in hepatocytes and trophoblastic cells. 1–4. The active stage. After bacterial internalization into the host cell and transient residence within a primary vacuole (**1**), bacteria escape into the cytoplasm, multiply and induce expression of the actin-polymerizing factor ActA (**2**). Actin recruitment and polymerization promotes bacterial motility **(3)** and cell-to-cell spread via the generation of membrane protrusions from the primary infected cell to neighbor cells (**4)**. After resolution of the protrusions (**5**), bacteria are in double-membrane secondary vacuoles, from which they escape and start a new cycle of infection **(6)**. **7–8. The phenotypic switch**. During the dissemination stage, bacteria stop producing ActA by an unknown asynchronous mechanism. ActA-free cytosolic bacteria (beige bacteria) multiply in the cytosol (**7**). A xenophagy-like process captures actin-free cytosolic bacteria into tertiary *Listeria*-containing vacuoles (LisCV) (**8**). **9. The LisCV stage**. LisCVs are lysosome-like compartments, in which subpopulations of bacteria resist stress and degradation and enter a slow/non-replicative state (dark orange bacteria), others are sensitive to stress and die (white bacteria with a “*”), and others enter a VBNC state (yellow bacteria). **10–11. The reactivation stage**. Unidentified stimuli induce reactivation of bacteria, which exit from the vacuoles (**10**). Production of ActA at the bacterial surface re-initiates a novel cycle of actin-polymerization and spreading (**11**). **12–14. Behavior of ActA-deficient bacteria**. *actA* mutants escape from the primary vacuole, replicate in the cytosol (**12**) and are captured by a xenophagy-like process (**13**). In LisCVs, bacteria enter a slow/non-replicative state (dark orange), or die (*) or enter a VBNC state (yellow) (**14**). **15. The dormant stage**. Δ*actA* bacteria are propagated during host cell divisions as VBNC contaminants. In absence of a reactivation signal, wild type bacteria may behave similarly to Δ*actA* bacteria and acquire the VBNC state. **16**. Gentamicin selects vacuolar and VBNC *L*. *monocytogenes* by inhibiting the growth of cytosolic bacteria.

This study presents the first *in vitro* model of intracellular persistence of *L*. *monocytogenes* in human cells, and particularly describes a vacuolar phase in the infectious process of *L*. *monocytogenes* in hepatocytes and placental cells (Fig 8). During intercellular dissemination, bacteria non-synchronously stop producing ActA and polymerizing actin. Actin-free cytosolic bacteria are engulfed in lysosome-like vacuoles, where they enter a slow/non-replicative state. Bacteria have the capacity to exit this resting phase when the host cells are subcultured. However, if the ActA deficiency is maintained, a bacterial population survives in a quiescent state known as VBNC. The antibiotic gentamicin selects vacuolar persistent forms of *L*. *monocytogenes* by eliminating cytosolic bacteria. This *in vitro* model should be valuable to study the molecular determinants of the asymptomatic carriage *L*. *monocytogenes* in epithelial tissues and to establish tests for the detection of dormant *Listeria* in clinical samples.

### *Listeria* life in vacuoles

Several species of bacterial pathogens proliferate in the cytosol of mammalian cells and use actin-based motility to propagate from cell to cell [8]. *L*. *monocytogenes* is one of the most studied species of cytosolic bacterial pathogens. However, the intracellular lifecycle of this bacterium needs to be revisited in light of studies showing that *L*. *monocytogenes* evolved means of living in vacuoles. The initially described mechanism takes place in murine macrophages, where a subset of bacteria remains confined in non-degradative phagosomes, known as SLAPs [20, 21, 39]. Here, we report another mechanism leading to the formation of *Listeria*-containing vacuoles (LisCVs), which could represent an adaptive strategy for this pathogen to establish long-term quiescent infections in epithelial tissues. In contrast to SLAPs, which are coupled to phagocytosis at the onset of infection, LisCVs are formed at a later stage (48h-72h) following the engulfment of actin-free bacteria by endomembranes. In addition, SLAPs are not acidic compartments, in contrast to LisCVs that display lysosomal features.

The segregation of cytosolic bacteria into LisCVs may occur by a mechanism related to xenophagy, a selective autophagy process that restricts the growth of intracellular microbes [44, 54]. Intracellular pathogens have evolved several strategies to bypass or subvert xenophagy and proliferate in host cells [54–59]. In particular, *L*. *monocytogenes* uses various mechanisms to evade killing by xenophagy at the early onset of an intracellular infection, such as manipulation of the mTOR pathway [54], stalling of the phagophores by bacterial phospholipases [15, 60] and camouflage of the bacteria by surface proteins, particularly by ActA [14, 15, 38]. While xenophagy avoidance is an important means for *L*. *monocytogenes* to promote its intracellular growth, hijacking the host autophagy machinery might also promote intracellular persistence, as suggested for SLAPs [39]. However, although LisCVs are formed by the entrapment of cytosolic bacteria in membrane-bound compartments, we did not find any evidence that supports a role of the canonical autophagy machinery in the formation of these vacuoles. It is interesting to note that the cytosolic pathogen *Mycobacterium marinum* can also be captured in vacuoles with lysosomal characteristics by a mechanism independent of canonical autophagy [61]. LisCVs could therefore be formed through an uncharacterized immune process that allows host cells to sequester microbial invaders from the cytosol. Future work will aim at characterizing the mechanism of LisCVs formation as well as its relevance to other infectious diseases.

### Emergence of VBNC *L*. *monocytogenes* during intracellular infection

LisCVs contain mixed populations of stress-sensitive and stress-resistant bacteria, as evidenced by the coexistence of damaged and intact bacteria. The latter survive the harsh conditions of lysosome-like organelles, like the intravacuolar pathogen *Coxiella burnetii* [62]. This resistance probably results from the ability of *L*. *monocytogenes* to withstand various stresses, including low pH conditions [63]. Many stresses trigger the VBNC state of bacteria [50]. Here, we provide the first evidence that *L*. *monocytogenes* can enter a VBNC state during infection, a property that had only been observed for *Listeria* in environmental conditions [64–67]. This conclusion is based on the phenotype of *actA* mutants, which model the behavior of *Listeria* unable to switch back to a motile state. *L*. *monocytogenes* VBNC bacteria may represent a dormant form of intracellular parasites that could be passively propagated during mitosis or by other mechanisms, such as direct cytosolic transfer between two adjacent cells [68]. Importantly, wild type *L*. *monocytogenes* retain the ability to exit from LisCVs and return to the active proliferation and dissemination phase upon subculture of host cells. We suggest that this phenotypic switching allows *L*. *monocytogenes* to disseminate or hide in tissues during the acute or asymptomatic phases of the infection, respectively. The identification of the molecular determinants required for the transition to the VBNC state during infection could lead to the development of therapeutics that target asymptomatic infections.

### Antibiotic tolerance and pathogenic potential of persistent forms of *L*. *monocytogenes*

Vacuolar pathogens may persist in the host for long periods, sometimes for the entire life of the host. For instance, the persistence of *Mycobacterium tuberculosis* in lungs, designated as “latency” or “dormancy”, is responsible for the silent carriage of this pathogen by nearly two billion people worldwide [69, 70]. *L*. *monocytogenes* is not described as a pathogen leading to subclinical infections, but it cannot be excluded that this bacterium might hide and persist in tissues. It has been shown that high-risk factors for sporadic non-perinatal listeriosis are mainly immunosuppressive diseases rather than the ingestion of particular foods [71]. In light of our results, the reactivation of dormant intracellular *L*. *monocytogenes*, long after the ingestion of contaminated foods, should be examined as a potential cause of sporadic listeriosis. The existence of a dormant subpopulation of *L*. *monocytogenes* might also be involved in recurrent listeriosis [72–74] and recurrent spontaneous miscarriages during the first weeks of pregnancy [75]. Moreover, not only silent infection may affect humans, but also animals. The phenotype described here may have relevance for animal reservoirs that could be ignored because of an asymptomatic intracellular carriage of *L*. *monocytogenes*.

Entry into an intracellular persistent state may promote tolerance of *L*. *monocytogenes* to antibiotic treatment during infection, as shown for several bacterial pathogens [76, 77]. The occurrence of non-replicating bacteria that survive exposure to antibiotics is proposed to account for the ability of pathogens to cause difficult-to-treat infections. Here, we found that gentamicin alters the intracellular growth of cytosolic *L*. *monocytogenes* but does not seem to act on vacuolar *Listeria*. The acidic LisCV could be a niche where *Listeria* is protected against gentamicin and other antibiotics. It is of note that aminoglycosides, such as gentamicin, are almost devoid of activity at the acidic pH of lysosomes [78]. Bacteria found within LisCVs might also share characteristics with small-colony variants, which are tolerant to aminoglycosides [79], and/or with *Listeria* persisters that emerge during bacterial growth in cell-free broth supplemented with bactericidal antibiotics [80]. The results presented here should have a significant clinical impact since the primary treatment for listeriosis consists of a combination regimen of gentamicin and ampicillin [81]. Beside tolerance to antibiotics, it is possible that cells carrying dormant *L*. *monocytogenes* are not effectively presenting *Listeria* antigens and are not efficiently recognized and destroyed by immune cells.

The physiological relevance of vacuolar forms of *L*. *monocytogenes* in epithelial tissues now needs to be demonstrated by *in vivo* studies. It is worth mentioning that hepatocytes of mice infected for 24h with *L*. *monocytogenes* mostly contain actin-free cytosolic bacteria [82], which could be in the early process of being sequestered in LisCVs. However, due to the low internalization rate of *Listeria* in murine epithelial cells [24] and to differences in mucosal immunity between mice and humans [25], the murine model of listeriosis may not be optimal for investigating the persistence of *L*. *monocytogenes* in epithelial tissues. Moreover, asymptomatic persistent forms of *Listeria* are likely to be in a very low number in tissues and difficult to observe. In conclusion, there is a need to develop animal models and easy-to-use diagnostic methods for the detection and study of VBNC *Listeria* in tissue samples. The possibility that *L*. *monocytogenes* persistent forms could be tolerant to antibiotics and undetectable by routine culture methods would clearly be a concern for public health safety assessment.

## Materials and methods

### Ethics statement

Primary human hepatocytes were purchased from Life Technologies. These liver cells were derived from tissue obtained from accredited institutions. These institutions obtained consent from the donors for use of the tissue and its derivative for research purpose.

### Bacterial strains and human cells

We used *L*. *monocytogenes* wild type strains EGDe (BUG1600, ATCC BAA-679) [32], 10403S [83] and CLIP 63713 (BUG1559, [34]), and mutant strains 10403S-Δ*hly* (DP-L2161) [84] and 10403S-Δ*actA* (DP-L1942, BUG1362 [83]). EGDe-Δ*actA* (BUG2167) was generated by allelic exchange, as described previously [85]. The resulting in-frame deletion mutant lacking the region encoding the ActA protein was verified by DNA sequence analysis of the chromosomal deletion. BUG2167 was used to construct EGDe-Δ*actA+actA*, which is described in [52]. Fluorescence-expressing strains of *L*. *monocytogenes* were 10403S-mCherry [86], EGDe-GFP (BUG2538) [87] and 10403S-GFP, EGDe-Δ*actA-*GFP and 10403S-Δ*actA-*GFP (HBSC43, HBSC155 and HBSC44, respectively), which were generated by chromosomal integration of plasmid pAD1-cGFP, as described previously [87]. Bacterial strains were grown at 37°C in brain heart infusion (BHI) broth (with agitation) or on BHI agar plates. Human cell lines were HepG2 hepatocytes (ATCC HB-8065), Huh7 hepatocytes (Japan Health Science Research Resources Bank, JCRB0403), HeLa epithelial cervix cells (ATCC CCL-2.2), JEG3 and BEWO trophoblast cells (ATCC HTB-36 and ATCC CCL-98), and HEK293 embryonic kidney cells (ATCC CRL 1573). Cells were grown under standard cell-culture conditions following the American Type Culture Collection (ATCC) recommendations. Human primary hepatocytes were cryopreserved Human Plateable Hepatocytes Metabolism Qualified (HMCPMS) from three human donors (lots HU1530, HU1583, HU8272, quality certificated by Life Technologies). Hepatocytes were thawed in thawing medium (CM7000, Life Technologies), seeded in 24-well Collagen I-coated plates (A1142802, Life Technologies) at 2.5x10^5^ cells/ml in Williams’ Medium E supplemented with 10% FBS, using supplement hepatocyte maintenance and plating supplement packs (CM3000, Life Technologies) and protocol provided by the supplier. Hepatocytes were infected after 48h of growth.

### Antibodies, reagents, transfection and RT-QPCR

The primary antibodies used in this study were against *L*. *monocytogenes* (polyclonal antibodies R11 or R97 [88]), ActA (polyclonal antibody R32 [89] or monoclonal antibody A16 [90]), LAMP1 (PE Mouse Anti-Human CD107a, BD Bioscience #555801; mouse antibody H4A3, Abcam ab25630), cathepsin D Goat polyclonal antibody (C-20, SantaCruz, sc-6486) and LC3 (clone 4E12, 152–3 MBL or clone 2G6, Nanotools). Fluorescent secondary antibodies were Alexa Fluor 488-conjugated (Life Technologies), Cy3-conjugated and Cy5-conjugated (Jackson ImmunoResearch Laboratories) goat anti-mouse or anti-rabbit antibodies, or Donkey anti-mouse or anti-rabbit antibodies. We also used Alexa fluor 488-, 568 or 647-conjugated phalloidin (Life Technologies), DAPI (Roche Applied Sciences), Hoechst (Thermo Fisher Scientific) and mounting media Fluoromount-G (Interchim, Montlucon). Gentamicin was from Sigma. pCBD-YFP plasmid (BUG2305) is described in [37]. pEGFP-LC3 plasmid (BUG 3046) is described in [38]. siRNAs were Silencer select siRNA ATG7 (IDS20650) and BECN1 (IDS16537) as described in [43]. pCBD-YFP or siRNAs were transiently transfected in cells with Lipofectamine LTX or Lipofectamine RNAiMax (Life Technologies), respectively, according to the manufacturer's instruction. pCBD-YFP was transfected 24h prior to infection. pEGFP-LC3 was transfected either 6h prior infection and infected for 3 days, or transfected after 1 day of infection and incubated for 2 other days. Both experimental procedures gave the same results. For siRNA assays, infected cells were submitted to two successive siRNA treatments, at 24h and 48h p.i. and fixed or processed for CFU counts or RNA extraction at 72h p.i. Procedures for RNA extraction and quantification by Real-Time qPCR, and *GAPDH* and *YWHAZ* PCR primers, are described in [91]. PCR primers for *ATG7* are Atg7-Fw, 5’-GATCCGGGGATTTCTTTCACG-3’ and Atg7-Rv, 5’-CAGCAATGTAAGACCAGTCAAGT-3’, and for *Beclin1*, BECN1-Fw 5’-GGCTGAGAGACTGGATCAGG-3’ and BECN1-Rv 5’-CTGCGTCTGGGCATAACG-3’.

### Bacterial infections, bacterial viability and LysoTracker assays

Human cells were seeded into 6- or 24-well plates (with or without coverslips) 2–4 days prior infection in order to reach 50–80% confluency (for all cell lines except JEG3, and for transfection assays) or 90–100% confluency (JEG3 monolayer assays). A detail protocol for studying LisCVs in JEG3 cell monolayers is available in *protocol*.*io* (dx.doi.org/10.17504/protocols.io.kwmcxc6). Bacterial colonies from freshly streaked agar plates were cultured in BHI broth and incubated overnight at 37°C in a shaking incubator. Stationary-phase bacteria were washed once in PBS and diluted in culture medium without serum. After a washing step, cells were infected with bacteria at a multiplicity of infection (MOI) of 0.1 to 5 and centrifuged 2 min at 300g to synchronize the bacterial uptake. Cells were infected for 1h. When using MOI of 1–5, cells were exposed to a 10-min treatment with a medium containing 100 μg/ml gentamicin, which ensures that extracellular bacteria are rapidly killed. For the lower MOI of 0.1, this step can be skipped and replaced by two washing steps. Cells were washed once and subsequently incubated in complete medium with 25 μg/ml gentamicin. When indicated, lower concentrations of gentamicin were applied (0, 1 or 5 μg/ml). Cells were processed for immunofluorescence (see below) or lysed at various time points using cold distilled water. Viable bacterial counts of intracellular bacteria were determined by plating serial dilutions onto BHI agar and numbering colony-forming units (CFU). Cells were enumerated in parallel wells following trypsin detachment and trypan-blue staining. To distinguish live bacteria with intact membranes from dead bacteria with compromised membranes, the Live/Dead BacLight Bacterial Viability kit (Life Technologies) was used with a protocol adapted from [47] and [46] with some modifications. JEG3 cells infected with bacteria in 6-well plates containing coverslips were gently washed twice in MOPS/MgCl_2_ (0.1 M 3-(N-morpholino) propanesulfonic acid (MOPS), pH 7.4; 1 mM MgCl_2_). Then 1 ml of the Live/Dead staining solution (1.6 μM SYTO9, 20 μM Propidium Iodide, 0.1% Triton X100 in MOPS/MgCl_2._) was added to the wells and incubated for 15 min (10 min with centrifugation at 300g to prevent cell detachment and 5 min at room temperature in the dark). Cells were then gently washed in MOPS/MgCl_2_ solution. For staining acidic compartments, 1 ml of the infected cell medium was supplemented with 0.5 μl of LysoTracker (Life technologies) and 1 μl of Hoechst, mixed and added back to the well. Cells were incubated for 30 min at 37°C at 10% CO_2_, washed in MEM and incubated for 5 min in MEM in the dark. For both BacLight and LysoTracker assays, coverslips were mounted onto glass slides and sealed 2 min with clear nail polish and immediately examined under the microscope for a maximum of 10 minutes.

### Epifluorescence microscopy and quantifications

Cells containing coverslips were fixed in 4% paraformaldehyde (PFA) in 1X PBS for 30 min at room temperature, gently washed in 1X PBS, incubated in blocking solution (2% BSA in PBS, pH 7.4), or in methanol for 10 min on ice when using the cathepsin D antibody, permeabilized using 0.4% Triton X-100 in PBS for 4 min, washed three time in PBS and processed for immunofluorescence with the indicated antibody diluted in 2% BSA. Fluorescent phalloidin and DAPI (or Hoechst) were added with the secondary antibodies to label F-actin and nuclei, respectively. To discriminate intracellular from extracellular bacteria, a dual staining was performed, whereby extracellular bacteria were stained with anti-*Listeria* antibodies prior to cell permeabilization. Samples were mounted on glass coverslips and analyzed with fluorescent microscopes (Carl Zeiss Axiovert 135, AxioObserver.Z1, KEYENCE BZ-X710 or Yokogawa CSU-X1 spinning disk confocal system). Images were acquired with a 10x non-immersion objective, or 40x, 63x or 100x oil immersion objectives, and images were processed with Zen (Carl Zeiss), MetaMorph (Universal Imaging) or Image J softwares. For time-lapse microscopy, JEG3 cells were seeded in a 35mm dish (MatTek) and infected with 10403S-mCherry bacteria (MOI 0.1). Imaging was performed in media without phenol red containing ProLong Antifade Reagents for Live Cells (Life Technologies) and using the KEYENCE fluorescent microscope BZ-X710 equipped with an incubation chamber (37°C and 5% CO_2_). Images were acquired with a non-immersion 40x objective. To quantify the number of bacteria that associated with the indicated markers, as well as the number of nuclei, 10 to 50 microscopic fields were examined from coverslips of at least three independent experiments. Due to the frequent overlap of bacterial immunofluorescence signals, the number of bacteria per infected cell was estimated by the ratio (T/I)/C, where “T” is the total immunofluorescence signal of the area occupied by bacteria, “I” is mean intensity values of representative single bacteria and “C” is number of cells/nuclei is the area of interest.

### Cell sorting and subculturing of infected cells

For the FACS assays, HepG2 cells infected 3 days with GFP-expressing EGDe bacteria were trypsinized, washed once in PBS, resuspended in DMEM and subjected to cell sorting using a FACSAria II Cell Sorter (BD Bioscience). For each of three independent experiments, about 5x10^5^ GFP-positive cells were collected and seeded onto polylysine-coated 6-well plates (1x10^5^ cells per well) in complete medium containing 25 μg/ml gentamicin and were examined 1h, 8h or 3 days after FACS. For the propagation assays using trypsin detachment, cells were dissociated with trypsin (200 μl of Trypsin-EDTA solution, Life Technologies), counted, diluted 5-or 10-fold in complete medium supplemented with gentamicin 25 μg/ml and seeded in 6-well plates (containing or not coverslips). Cells were then incubated for the indicated time and either fixed to proceed to immunofluorescence assays, lysed with distilled water for CFU counting, or detached and subcultured again. It is of note that trypsinization and flow sorting may select for cell subpopulations. This is a limit of this assay.

### Transmission electron microscopy

First analytic experiments were performed at the Ultrapole platform of Institut Pasteur, as follows. Cells were fixed overnight with 2.5% Glutaraldehyde in 0.1M sodium cacodylate buffer at pH 7.2. Then samples were rinsed in 0.1M sodium cacodylate (pH 7.2) and post-fixed in a 1% osmium tetroxyde at room temperature with the same buffer for 1h. Samples were rinsed with water and dehydrated with a graded ethanol concentrations (10–100%), followed by a mixture of ethanol and embedded in an epoxy resin. Ultrathin sections (50–60 nm) were performed with an ultramicrotome « Ultracut UC7 » (Leica Microsystems, Vienna, Austria), stained with uranyl acetate and Reynold’s lead citrate, and then observed with a tecnai T12 (FEI company) at 100-kV accelerating voltage. Images were recorded using us 4000 gatan camera and digital micrograph software or Eagle camera and tia software. Quantitative experiments were performed at the MIMA2 platform (Jouy-en-Josas) or ImagGif platform (Gif-sur-Yvette), with similar procedures and the following modifications: cells were fixed overnight with 2% Glutaraldehyde, counter stained on block with 0.5% OTE (Oolong Tea Extract) in 0.1M sodium cacodylate buffer and post-fixed in a solution of containing 1% osmium tetroxyde and 1.5% potassium ferrocyanate; samples were rinsed with water and dehydrated with a graded 30–100% ethanol concentrations. Ultrathin sections were observed with Hitachi HT7700 or Jeol 1400 (Jeol—Allemagne) at 80-kV accelerating voltage.

## Supporting information

S1 Fig LisCVs are formed in a subset of epithelial cells. Cells were infected with the indicated *L*. *monocytogenes* strain (MOI ~ 1–5) and processed for epifluorescence microscopy at 72h p.i. The color of each staining is indicated on panel headlines. **A**. LAMP1-negative and Actin-positive EGDe bacteria in HeLa cells. Bar: 10 μm. **B**. LAMP1-positive EGDe bacteria in Huh7 hepatocytes. Bar: 5 μm. **C**. Strains EGDe (serotype 1/2a), 10403S (serotype 1/2a) and CLIP 63713 (serotype 4b) in JEG3 cells. Samples were labeled with monoclonal antibodies against LAMP1, polyclonal antibodies against *Listeria* serotype 1/2a (which do not labeled serotype 4b), phalloidin-Cy5 and DAPI. All strains stop polymerizing actin and become enclosed in LAMP1-positive compartments. Two magnifications are shown for each strain: on top panels, bars: 5 μm; on bottom panels, which highlight bacteria pointed by arrows, bars: 1 μm. The phase contrast images highlight intact bacilli. **D**. LAMP1-positive 10403S bacteria in BeWo cells. Bar: 20 μm. **E-G**. Primary human hepatocytes grown on collagen-coated plates were infected with *L*. *monocytogenes* 10403S bacteria (MOI ~ 5) and lysed at 2h and 72h p.i. to determine bacterial intracellular loads by CFU counts. **E**. The efficiency of bacterial entry in primary hepatocytes is compared to that in HepG2 hepatocytes or HeLa cells at the same MOI (~ 5) after 2h of infection. Results are mean±SD of triplicate experiments. **F**. Intracellular loads of 10403S bacteria in primary hepatocytes at 2h and 72h p.i. **G**. Micrographs of primary hepatocytes infected for 72h with 10403S. Overlays show *Listeria* (green), LAMP1 (red), F-actin (white) and DAPI (blue) signals. Bars: 5 μm. A high magnification of the region pointed with an arrow is shown below. Bar: 2 μm.
(TIF)

S2 Fig Structure of the *Listeria*-containing vacuoles. Transmission electron microscopy study of bacteria after three days of infection in JEG3 cells. These data are associated with Fig 1F and 1G. Each micrograph shows a representative stage of the *L*. *monocytogenes* cellular invasion process, as labeled in the black box on the left corner. The two micrographs labeled “pre-LisCV” highlight bacteria that might be in the process of being captured by electron-dense compartments. L.m, *L*. *monocytogenes*; F-actin, filamentous actin; Nuc., nucleus; Lys., secondary lysosomes; Mito., mitochondria; Mbs., membranous intravacuolar structures; Double mb. vacuole: secondary vacuole derived from a bacterial protrusion; LisCV: single-membrane *Listeria*-containing vacuole. Bars: 1 μm.
(TIF)

S3 Fig LisCVs are formed at a late stage of bacterial dissemination. **A**. JEG3 cells monolayers were infected for 72h with *L*. *monocytogenes* with 10403S or EGDe strain at MOI ~ 1 or ~ 0.1 and viable cells were numbered at different time points. **B-D**. Micrographs of cells infected with 10403S (MOI ~ 0.1) at low magnifications. **B**. At 2h p.i., bacteria were labeled with antibodies before (in red) and after (in green) cell permeabilization. Extracellular *Listeria* (both red and green) appear in yellow and intracellular *Listeria* in green. F-actin staining (in white) delimitate cell junctions (as exemplified for one cell with a dashed line). Bar: 20 μm. Bacteria pointed with arrows are shown at a higher magnification on the right (Bar: 5 μm). Images have been digitally processed to enhance the fluorescent signals in order to visualize each single bacterium. **C**. Micrographs of cells infected for 2, 6, 24 or 72h and visualized with the objective 10X. Images are overlays of *Listeria* (green) and F-actin (red) signals. Circles highlight an individual bacterium at 2h p.i., and an infection focus at 6h p.i. Bar: 100 μm. **D**. DAPI staining of non-infected (NI) and 10403S-infected JEG3 cells at 72h p.i. The arrows indicate altered nuclei. Bar: 100 μm. **E**. Intracellular growth of 10403S bacteria in JEG3 cells assessed by CFU counts (mean±SD of triplicate experiments). **F**. Quantification of 10403S bacteria in different phenotypes at 6h and 72h p.i (mean±SD of triplicate experiments).
(TIF)

S4 Fig LisCVs are formed after *L*. *monocytogenes* has passed through a cytosolic stage. JEG3 cells were transiently transfected with a plasmid encoding the *L*. *monocytogenes* cell-wall probe CBD-YFP and infected with *L*. *monocytogenes* 10403S (MOI ~ 0.1) for 6h, 24h and 72h. Samples were processed for epifluorescence microscopy. The micrographs are representative of results from three independent experiments. The color of each staining is indicated on panel headlines. Squared regions are shown at a higher magnification on the right (**A**), as well as below for 72h p.i. (**B**). Arrows point CBD-YFP dots at the surface of bacteria within LisCVs. Bars: 10 μm and 2 μm.
(TIF)

S5 Fig Long-term infection of JEG3 cell monolayers with *L*. *monocytogenes* 10403S-Δ*actA* bacteria. Micrographs of JEG3 cells infected with 10403S-Δ*actA* (MOI ~ 0.1) at low (on top) or high (on bottom) magnification. Images are overlays of *Listeria* (green), F-actin (red) signals. Circles highlight an individual bacterium at 2h p.i., and an infection focus at 72h p.i.
(TIF)

S6 Fig The canonical autophagy pathway is not necessary for the formation of LisCVs. **A-B**. JEG3 cells were infected with *Listeria* 10403S (MOI ~ 0.1) and processed for immunofluorescence with LC3 and *Listeria* antibodies at different time post-infection. **A**. The histograms represent the percentage of LC3-positive or LC3-negative bacteria (mean±SD of triplicate experiments). **B**. A representative image of the immunolabeling of LC3 and *Listeria* at 72h p.i. **C**. JEG3 cells expressing GFP-LC3 were infected with *Listeria* EGDe and processed for immunofluorescence assays with *Listeria* and LAMP1 antibodies. The arrows point regions, which are shown at a higher magnification below. Bars: 10 μm and 1 μm. **D-F** JEG3 cells were infected with 10403S bacteria, exposed to two successive siRNA treatments with *ATG7*, *BECN1* or control siRNAs, one at 24h p.i., and one at 48h p.i. At 72h p.i., samples were processed for transcript quantification (**D**), or immunofluorescence assays with LAMP1 and *Listeria* antibodies and DAPI (**E**), or CFU and JEG3 cell counting (**F**). In **D**, the fold change of transcript level is relative to values normalized to *GAPDH* reference gene. *YWHAZ* was used as a control gene.
(TIF)

S7 Fig LisCVs are stained by lysosomal markers. **A**. JEG3 cells monolayers were infected for 72h with the indicated strain and processed for microscopy analysis. The color of each staining is indicated on the panel headlines. **A**. Micrographs of live cells infected with GFP-expressing *L*. *monocytogenes* WT or Δ*actA* strains and stained with Lysotracker and Hoechst. Bars: 5 μm. Arrows point representative LysoTracker-positive LisCVs. **B**. Micrographs of fixed cells infected with EGDe-Δ*actA* or 10403S-Δ*actA* bacteria, and stained with *Listeria*, cathepsin D and LAMP1 antibodies. Arrows point representative cathepsin D-positive LisCVs. Bars: 2 μm.
(TIF)

S8 Fig Bacteria return to the replicative-motility phase in a subpopulation of cells after cell passaging. HepG2 cells containing EGDe bacteria entrapped in LisCVs at day 6 (as in Fig 5) were detached with trypsin, diluted to 2 x 10^5^ cell/mL and grown for 24h in complete medium with gentamicin 25μg/mL. The micrograph, which is representative of 5 experiments, is an overlay image of cells stained with *Listeria* and LAMP1 antibodies, fluorescent phalloidin to label F-actin and Hoechst to mark nuclei. Bar: 50 μm. Examples of cells in each of the two populations, either carrying persistent vacuolar *Listeria* (“P”) or carrying active Actin-associated *Listeria* (“A”), are framed and represented at a higher magnification below. Bar: 5 μm. The color of each staining is indicated on the panel headlines.
(TIF)

S9 Fig LAMP1^+^ bacteria are present in dividing host cells at different stages of mitosis. Cells were infected with EGDe-Δ*actA* and subcultured as in Fig 6A. At day 10, cells were processed for immunofluorescence with *Listeria* and LAMP1 antibodies and DAPI. LAMP1^+^ compartments containing bacteria are pointed in cells at different steps of mitosis. Micrographs are representative of six independent experiments. Bars: 10 μm.
(TIF)

S10 Fig Re-expression of *actA* in the EGDe-Δ*actA* mutant promotes the motile stage of *L*. *monocytogenes*. JEG3 cells were infected with *L*. *monocytogenes* EGDe or EGDe-Δ*actA+actA* strains for three days and stained with DAPI, ActA antibodies and 647-conjugated-phalloidin to label F-actin. Bar: 10 μm.
(TIF)

S11 Fig The *L*. *monocytogenes* phenotypic switch occurs in cells grown with a low concentration of gentamicin. Representative micrographs of JEG3 cells infected with *L*. *monocytogenes* EGDe (MOI ~ 0.1; without 10-min exposure to gentamicin 100 μg/mL) for 72h in presence of gentamicin 5 or 25 μg/mL. Cells were labeled with *Listeria* polyclonal antibodies (green in the overlay), LAMP1 (A) or ActA (B) monoclonal antibodies (red in the overlay) and Hoechst (Blue in the overlay). White arrows point LisCVs. Bar: 10 μm.
(TIF)

S12 Fig Variability of the phenotype of 10403S-Δ*actA* bacteria in JEG3 cells subcultured in gentamicin 5 μg/mL for 13 days. JEG3 cells infected with *L*. *monocytogenes* 10403S-Δ*actA* (MOI ~ 1) and propagated up to d13 in presence of gentamicin 5 or 25 μg/mL. A 12 mm coverslip placed in the well was recovered just before lysing the cells, in order to process the same cells for immunofluorescence and CFU counts. Micrographs show representative cells from (**A)** wells leading to VBNC bacteria (not detectable colony, “nd”) or (**B**) bacteria forming colonies (1.3 10^5^ CFU/well). Cells were labeled with *Listeria* polyclonal antibodies (green), LAMP1 monoclonal antibodies (red), fluorescent phalloidin (white) and Hoechst (Blue). White arrows point LAMP1^+^ bacteria. Bar: 5 μm.
(TIF)

S1 Movie Time-lapse micrographs of JEG3 cells infected with mCherry-expressing 10403S. Cells were infected as described in the Materials and Methods section. Bright field and fluorescent signals were obtained from 5 Z-positions covering 5 μm of thickness at 1h interval using an autofocus system. Best focus images were manually selected and post-treated. Three representative cells that show formation of LisCVs are pointed with arrows. Time is indicated in the upper right corner (h:m). Scale bar is 50 μm.
(MOV)
