# Supplementary figures and images for: Listeria monocytogenes switches from dissemination to persistence by adopting a vacuolar lifestyle in epithelial cells
Source: PLoS Pathog. 2017 Nov 30;13(11):e1006734. doi: 10.1371/journal.ppat.1006734 (PMC5708623; doi:10.1371/journal.ppat.1006734)

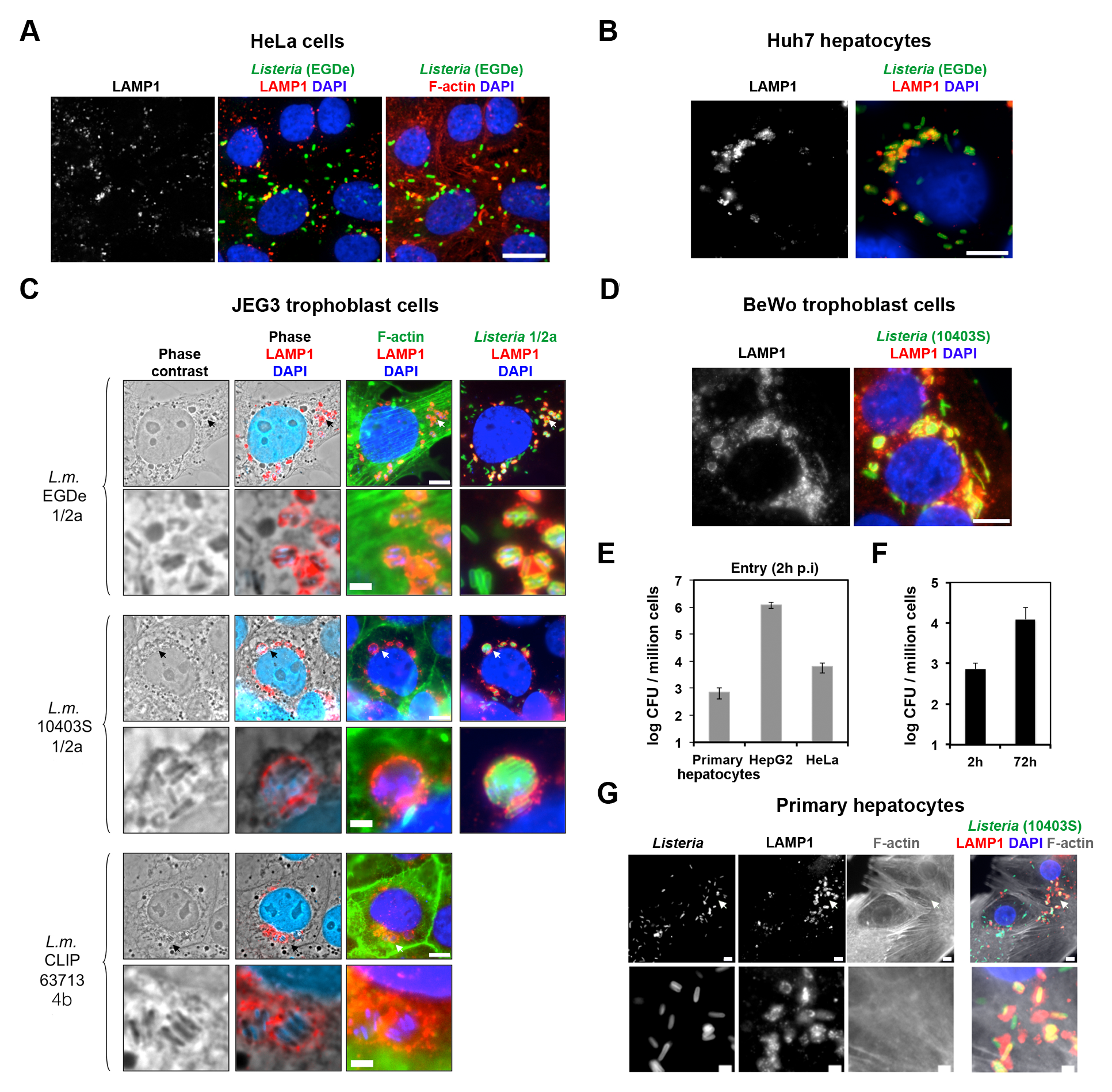

Supplement: S1 Fig — Cells were infected with the indicated L. monocytogenes strain (MOI ~ 1–5) and processed for epifluorescence microscopy at 72h p.i. The color of each staining is indicated on panel headlines. A. LAMP1-negative and Actin-positive EGDe bacteria in HeLa cells. Bar: 10 μm. B. LAMP1-positive EGDe bacteria in Huh7 hepatocytes. Bar: 5 μm. C. Strains EGDe (serotype 1/2a), 10403S (serotype 1/2a) and CLIP 63713 (serotype 4b) in JEG3 cells. Samples were labeled with monoclonal antibodies against LAMP1, polyclonal antibodies against Listeria serotype 1/2a (which do not labeled serotype 4b), phalloidin-Cy5 and DAPI. All strains stop polymerizing actin and become enclosed in LAMP1-positive compartments. Two magnifications are shown for each strain: on top panels, bars: 5 μm; on bottom panels, which highlight bacteria pointed by arrows, bars: 1 μm. The phase contrast images highlight intact bacilli. D. LAMP1-positive 10403S bacteria in BeWo cells. Bar: 20 μm. E-G. Primary human hepatocytes grown on collagen-coated plates were infected with L. monocytogenes 10403S bacteria (MOI ~ 5) and lysed at 2h and 72h p.i. to determine bacterial intracellular loads by CFU counts. E. The efficiency of bacterial entry in primary hepatocytes is compared to that in HepG2 hepatocytes or HeLa cells at the same MOI (~ 5) after 2h of infection. Results are mean±SD of triplicate experiments. F. Intracellular loads of 10403S bacteria in primary hepatocytes at 2h and 72h p.i. G. Micrographs of primary hepatocytes infected for 72h with 10403S. Overlays show Listeria (green), LAMP1 (red), F-actin (white) and DAPI (blue) signals. Bars: 5 μm. A high magnification of the region pointed with an arrow is shown below. Bar: 2 μm. (TIF) [file ppat.1006734.s001.tif]

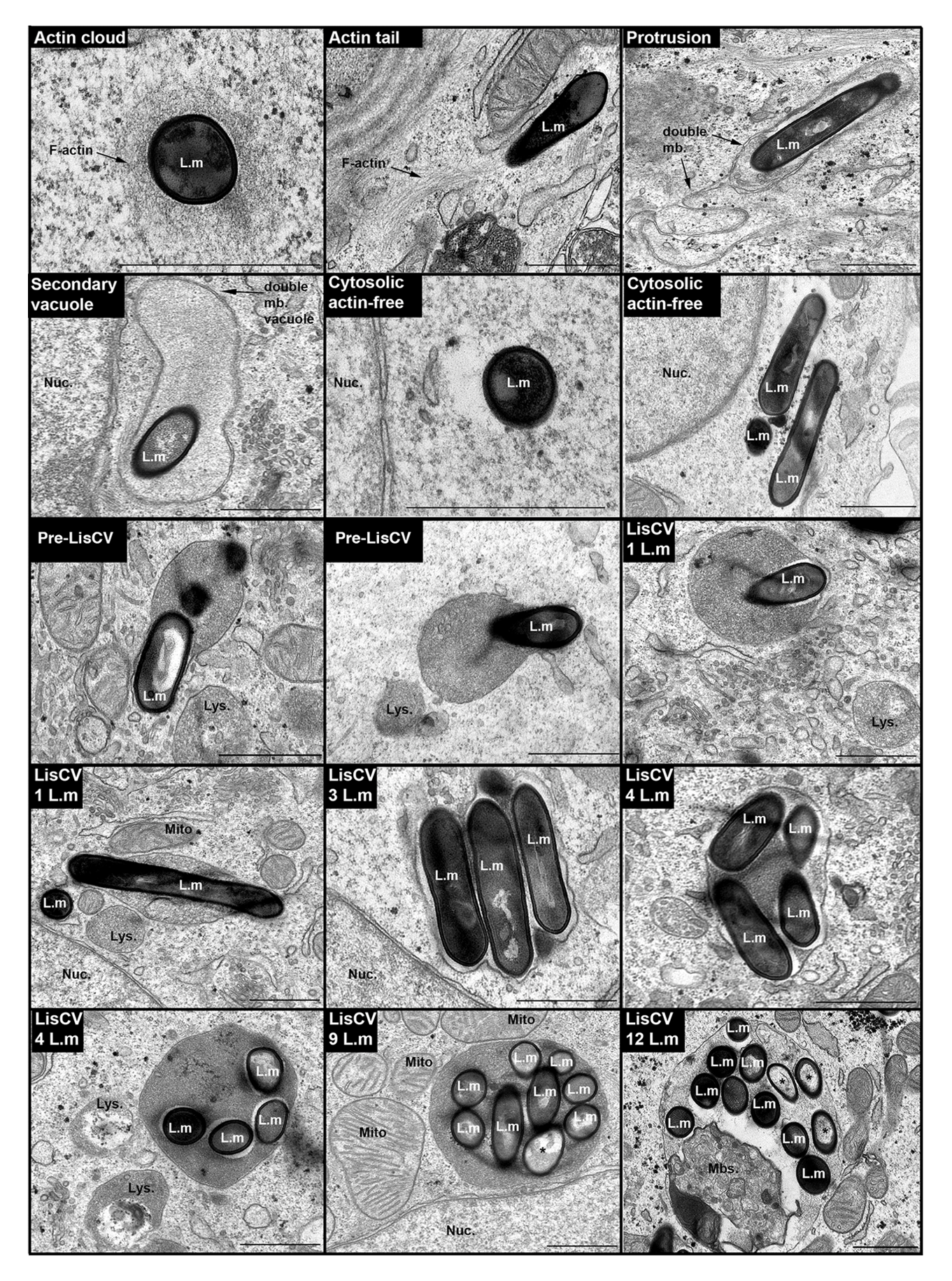

Supplement: S2 Fig — Transmission electron microscopy study of bacteria after three days of infection in JEG3 cells. These data are associated with Fig 1F and 1G. Each micrograph shows a representative stage of the L. monocytogenes cellular invasion process, as labeled in the black box on the left corner. The two micrographs labeled “pre-LisCV” highlight bacteria that might be in the process of being captured by electron-dense compartments. L.m, L. monocytogenes; F-actin, filamentous actin; Nuc., nucleus; Lys., secondary lysosomes; Mito., mitochondria; Mbs., membranous intravacuolar structures; Double mb. vacuole: secondary vacuole derived from a bacterial protrusion; LisCV: single-membrane Listeria-containing vacuole. Bars: 1 μm. (TIF) [file ppat.1006734.s002.tif]

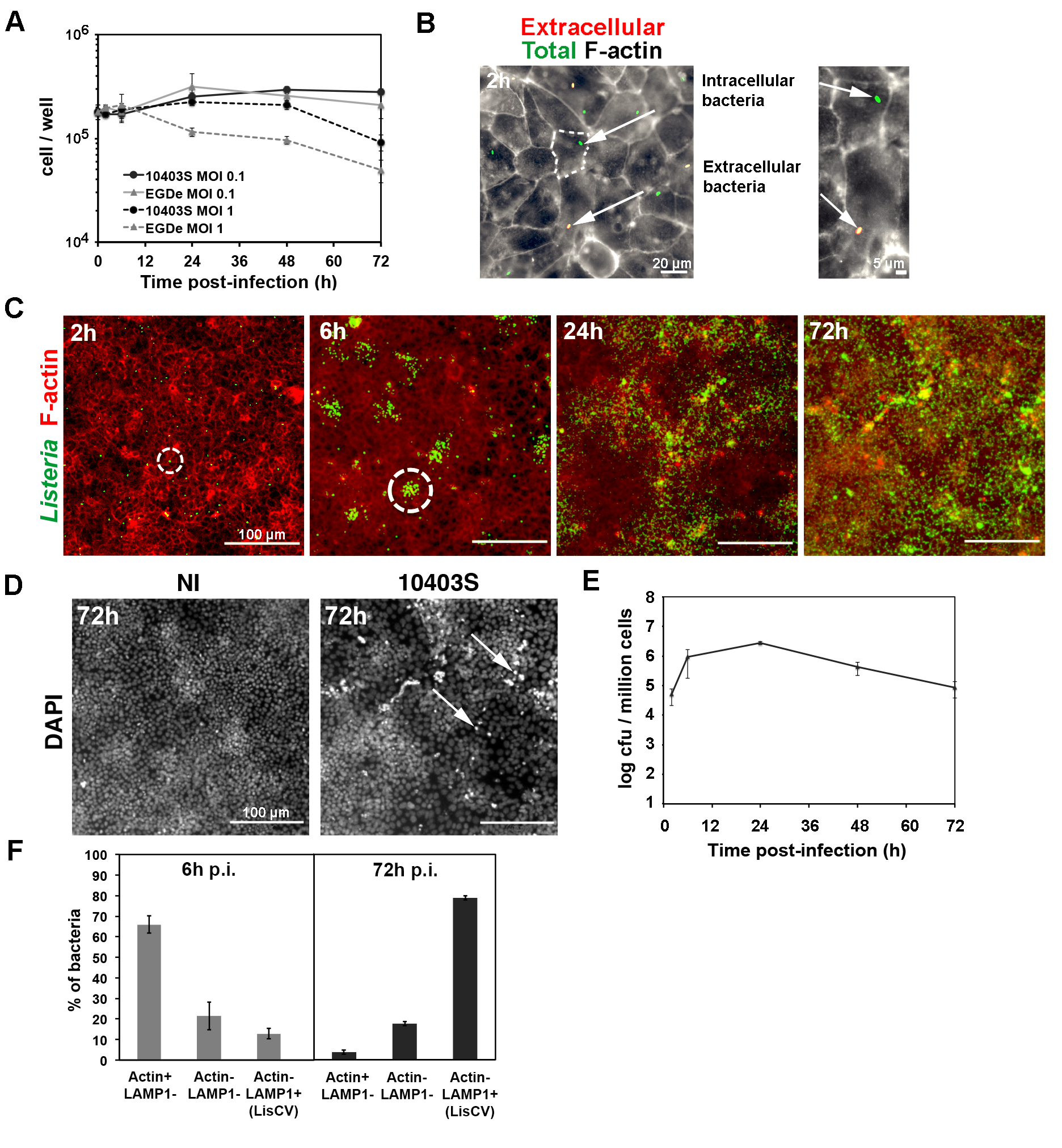

Supplement: S3 Fig — A. JEG3 cells monolayers were infected for 72h with L. monocytogenes with 10403S or EGDe strain at MOI ~ 1 or ~ 0.1 and viable cells were numbered at different time points. B-D. Micrographs of cells infected with 10403S (MOI ~ 0.1) at low magnifications. B. At 2h p.i., bacteria were labeled with antibodies before (in red) and after (in green) cell permeabilization. Extracellular Listeria (both red and green) appear in yellow and intracellular Listeria in green. F-actin staining (in white) delimitate cell junctions (as exemplified for one cell with a dashed line). Bar: 20 μm. Bacteria pointed with arrows are shown at a higher magnification on the right (Bar: 5 μm). Images have been digitally processed to enhance the fluorescent signals in order to visualize each single bacterium. C. Micrographs of cells infected for 2, 6, 24 or 72h and visualized with the objective 10X. Images are overlays of Listeria (green) and F-actin (red) signals. Circles highlight an individual bacterium at 2h p.i., and an infection focus at 6h p.i. Bar: 100 μm. D. DAPI staining of non-infected (NI) and 10403S-infected JEG3 cells at 72h p.i. The arrows indicate altered nuclei. Bar: 100 μm. E. Intracellular growth of 10403S bacteria in JEG3 cells assessed by CFU counts (mean±SD of triplicate experiments). F. Quantification of 10403S bacteria in different phenotypes at 6h and 72h p.i (mean±SD of triplicate experiments). (TIF) [file ppat.1006734.s003.tif]

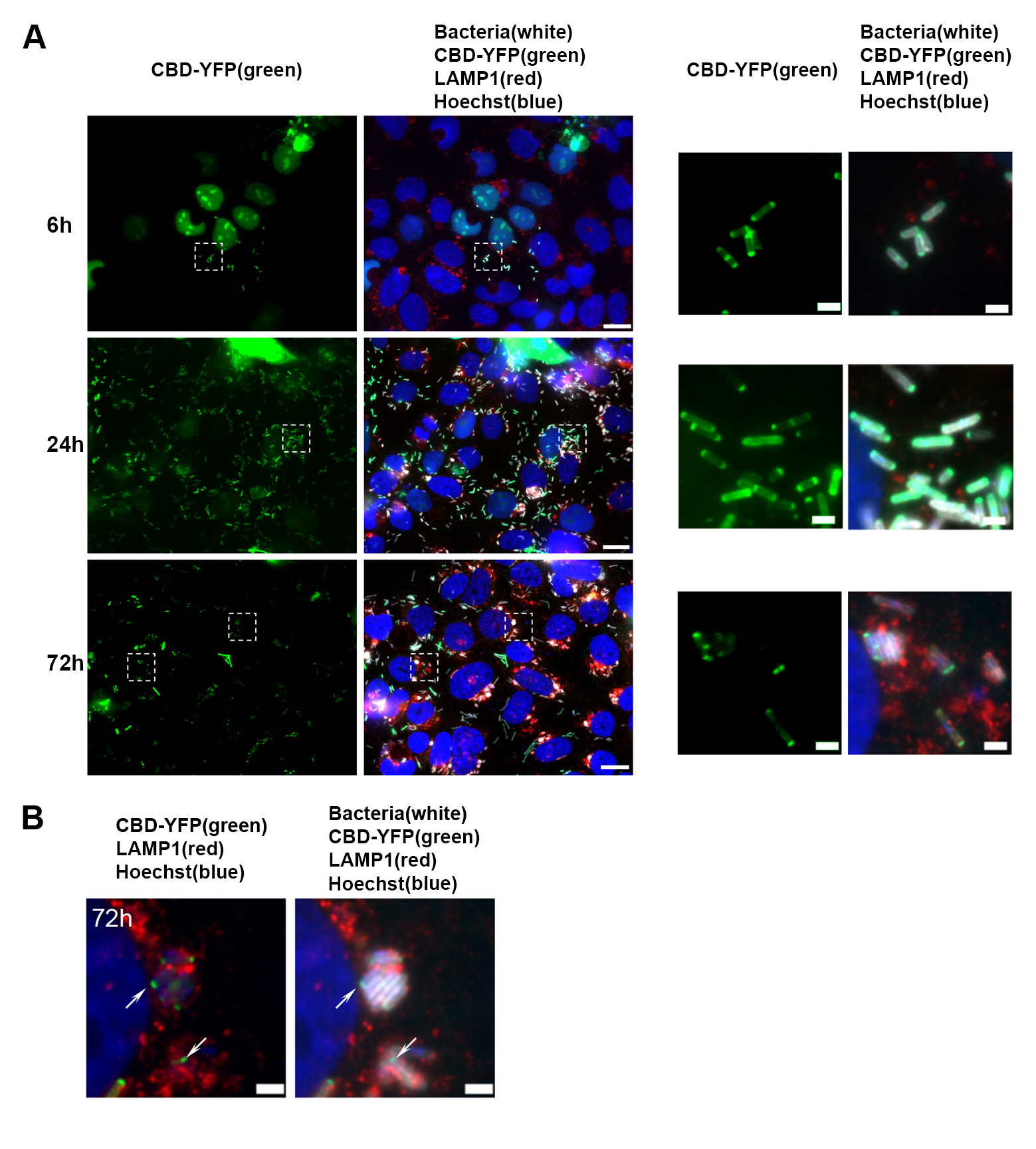

Supplement: S4 Fig — JEG3 cells were transiently transfected with a plasmid encoding the L. monocytogenes cell-wall probe CBD-YFP and infected with L. monocytogenes 10403S (MOI ~ 0.1) for 6h, 24h and 72h. Samples were processed for epifluorescence microscopy. The micrographs are representative of results from three independent experiments. The color of each staining is indicated on panel headlines. Squared regions are shown at a higher magnification on the right (A), as well as below for 72h p.i. (B). Arrows point CBD-YFP dots at the surface of bacteria within LisCVs. Bars: 10 μm and 2 μm. (TIF) [file ppat.1006734.s004.tif]

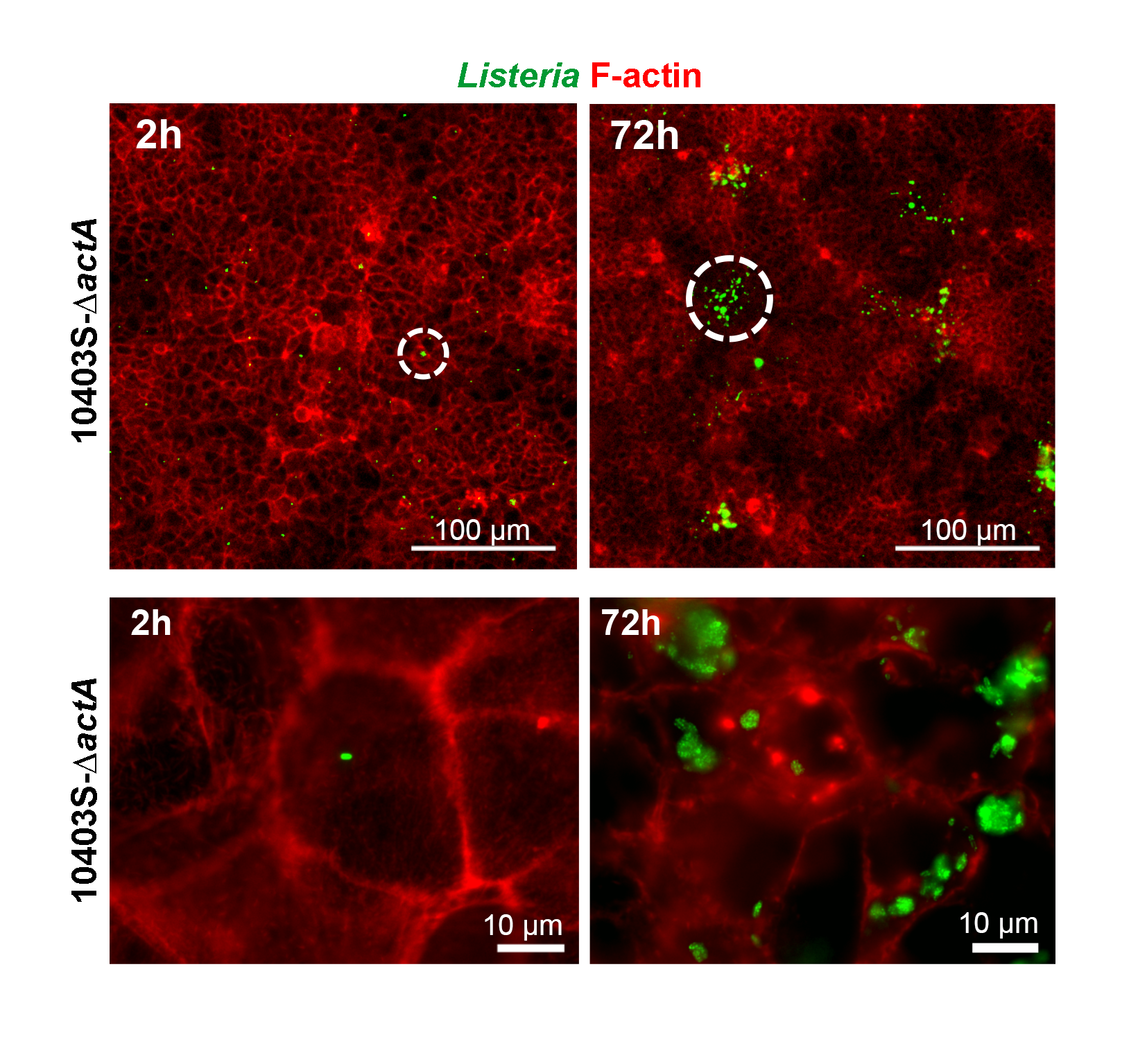

Supplement: S5 Fig — Micrographs of JEG3 cells infected with 10403S-ΔactA (MOI ~ 0.1) at low (on top) or high (on bottom) magnification. Images are overlays of Listeria (green), F-actin (red) signals. Circles highlight an individual bacterium at 2h p.i., and an infection focus at 72h p.i. (TIF) [file ppat.1006734.s005.tif]

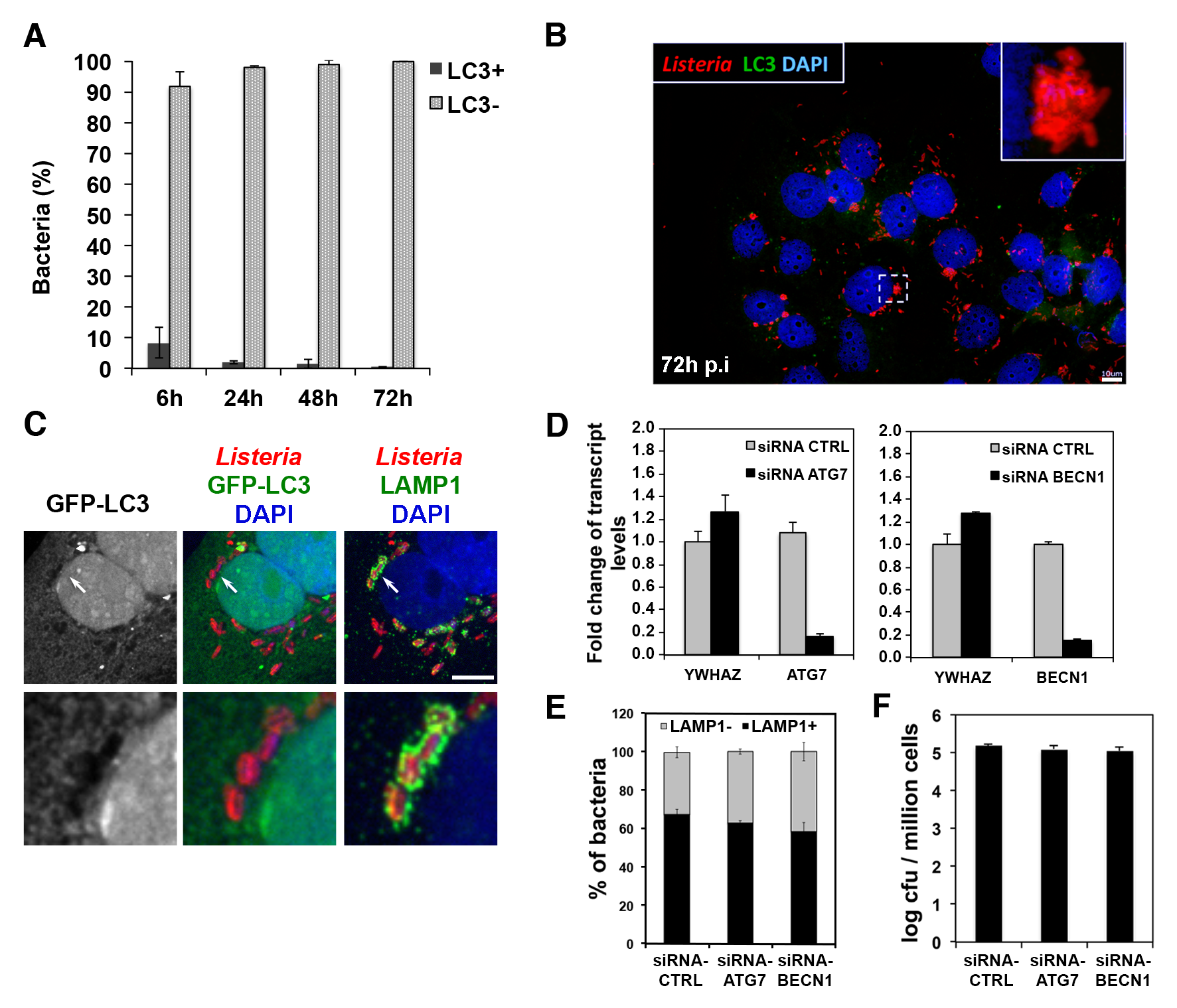

Supplement: S6 Fig — A-B. JEG3 cells were infected with Listeria 10403S (MOI ~ 0.1) and processed for immunofluorescence with LC3 and Listeria antibodies at different time post-infection. A. The histograms represent the percentage of LC3-positive or LC3-negative bacteria (mean±SD of triplicate experiments). B. A representative image of the immunolabeling of LC3 and Listeria at 72h p.i. C. JEG3 cells expressing GFP-LC3 were infected with Listeria EGDe and processed for immunofluorescence assays with Listeria and LAMP1 antibodies. The arrows point regions, which are shown at a higher magnification below. Bars: 10 μm and 1 μm. D-F JEG3 cells were infected with 10403S bacteria, exposed to two successive siRNA treatments with ATG7, BECN1 or control siRNAs, one at 24h p.i., and one at 48h p.i. At 72h p.i., samples were processed for transcript quantification (D), or immunofluorescence assays with LAMP1 and Listeria antibodies and DAPI (E), or CFU and JEG3 cell counting (F). In D, the fold change of transcript level is relative to values normalized to GAPDH reference gene. YWHAZ was used as a control gene. (TIF) [file ppat.1006734.s006.tif]

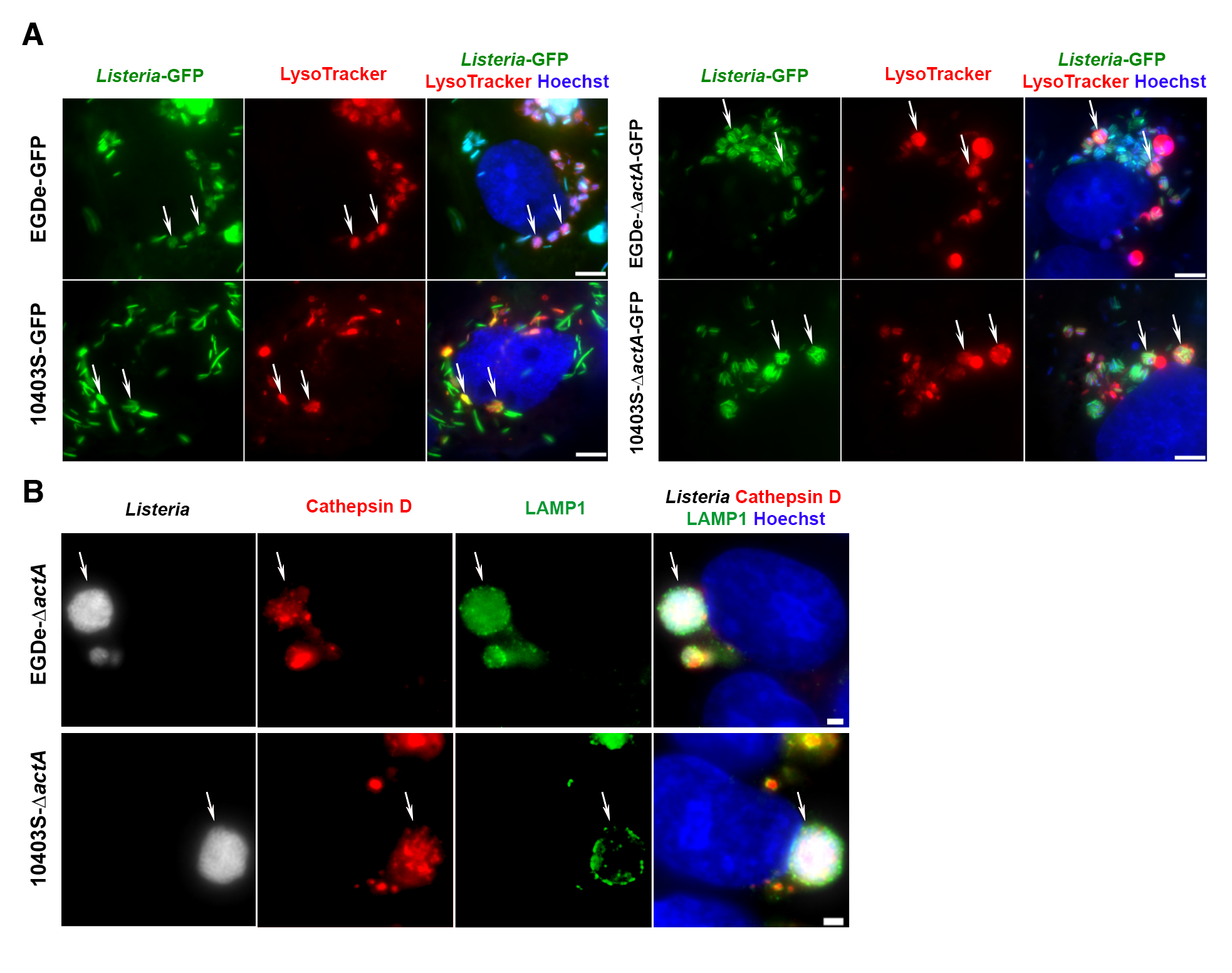

Supplement: S7 Fig — A. JEG3 cells monolayers were infected for 72h with the indicated strain and processed for microscopy analysis. The color of each staining is indicated on the panel headlines. A. Micrographs of live cells infected with GFP-expressing L. monocytogenes WT or ΔactA strains and stained with Lysotracker and Hoechst. Bars: 5 μm. Arrows point representative LysoTracker-positive LisCVs. B. Micrographs of fixed cells infected with EGDe-ΔactA or 10403S-ΔactA bacteria, and stained with Listeria, cathepsin D and LAMP1 antibodies. Arrows point representative cathepsin D-positive LisCVs. Bars: 2 μm. (TIF) [file ppat.1006734.s007.tif]

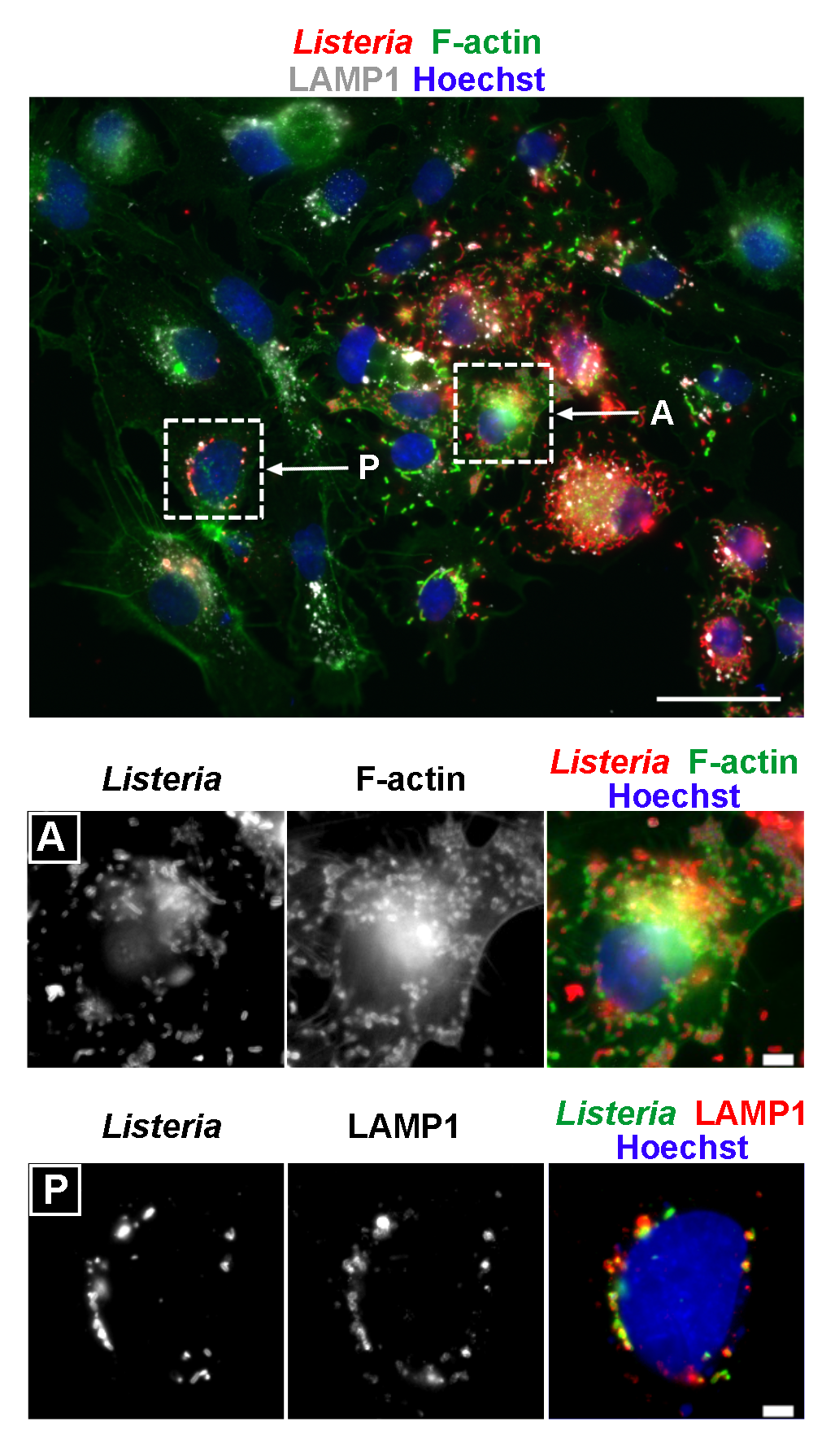

Supplement: S8 Fig — HepG2 cells containing EGDe bacteria entrapped in LisCVs at day 6 (as in Fig 5) were detached with trypsin, diluted to 2 x 10^5^ cell/mL and grown for 24h in complete medium with gentamicin 25μg/mL. The micrograph, which is representative of 5 experiments, is an overlay image of cells stained with Listeria and LAMP1 antibodies, fluorescent phalloidin to label F-actin and Hoechst to mark nuclei. Bar: 50 μm. Examples of cells in each of the two populations, either carrying persistent vacuolar Listeria (“P”) or carrying active Actin-associated Listeria (“A”), are framed and represented at a higher magnification below. Bar: 5 μm. The color of each staining is indicated on the panel headlines. (TIF) [file ppat.1006734.s008.tif]

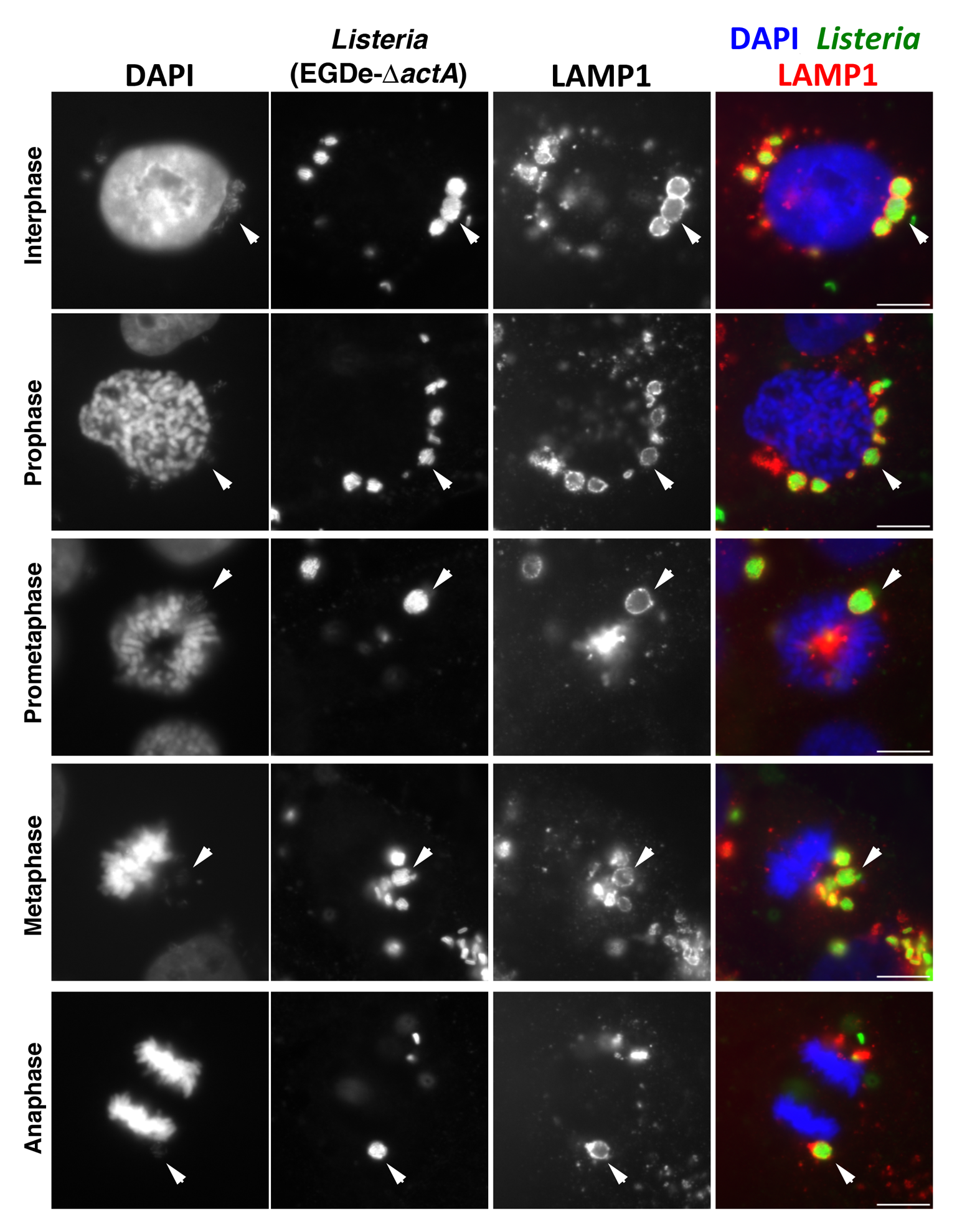

Supplement: S9 Fig — Cells were infected with EGDe-ΔactA and subcultured as in Fig 6A. At day 10, cells were processed for immunofluorescence with Listeria and LAMP1 antibodies and DAPI. LAMP1+ compartments containing bacteria are pointed in cells at different steps of mitosis. Micrographs are representative of six independent experiments. Bars: 10 μm. (TIF) [file ppat.1006734.s009.tif]

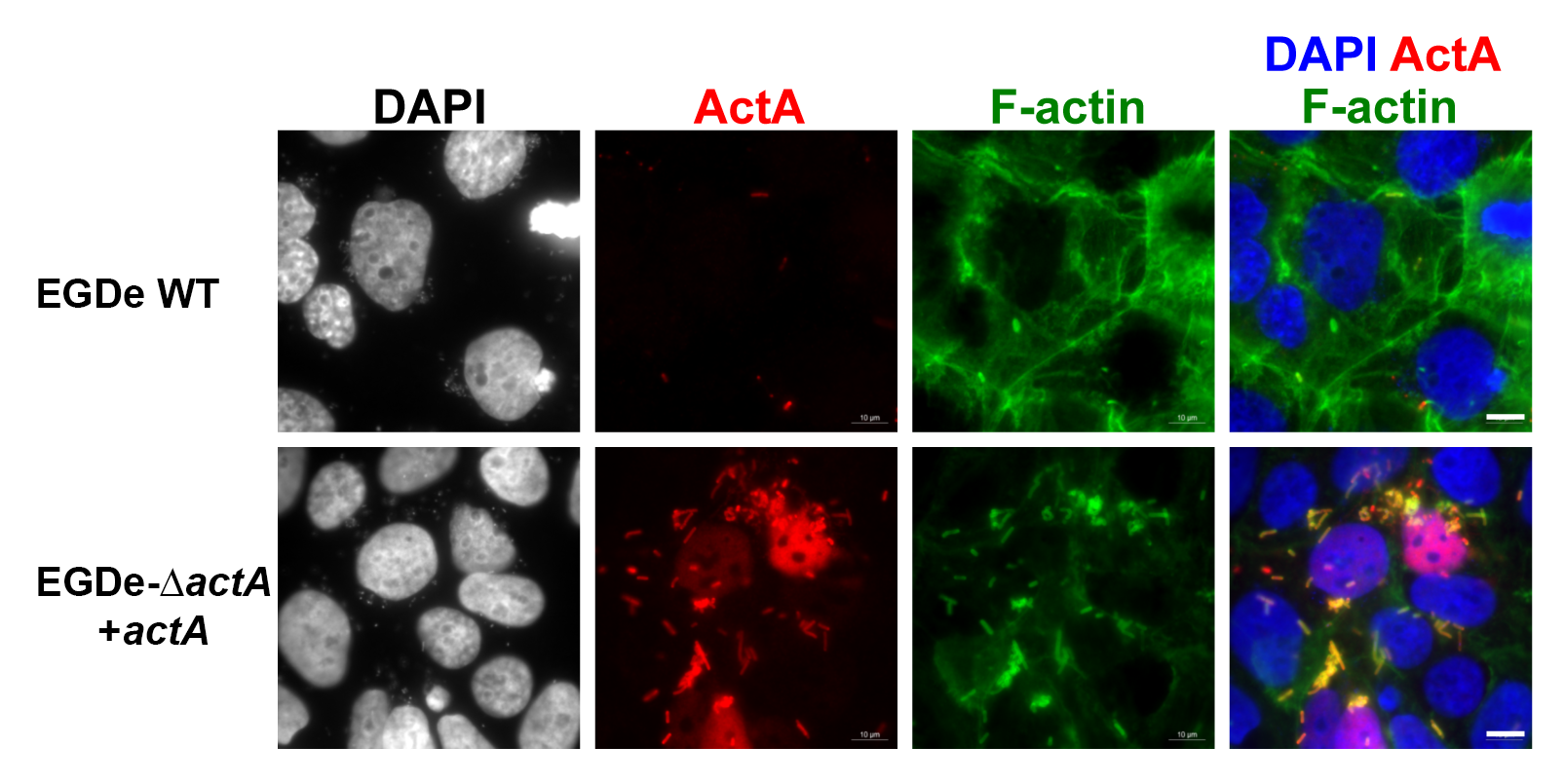

Supplement: S10 Fig — JEG3 cells were infected with L. monocytogenes EGDe or EGDe-ΔactA+actA strains for three days and stained with DAPI, ActA antibodies and 647-conjugated-phalloidin to label F-actin. Bar: 10 μm. (TIF) [file ppat.1006734.s010.tif]

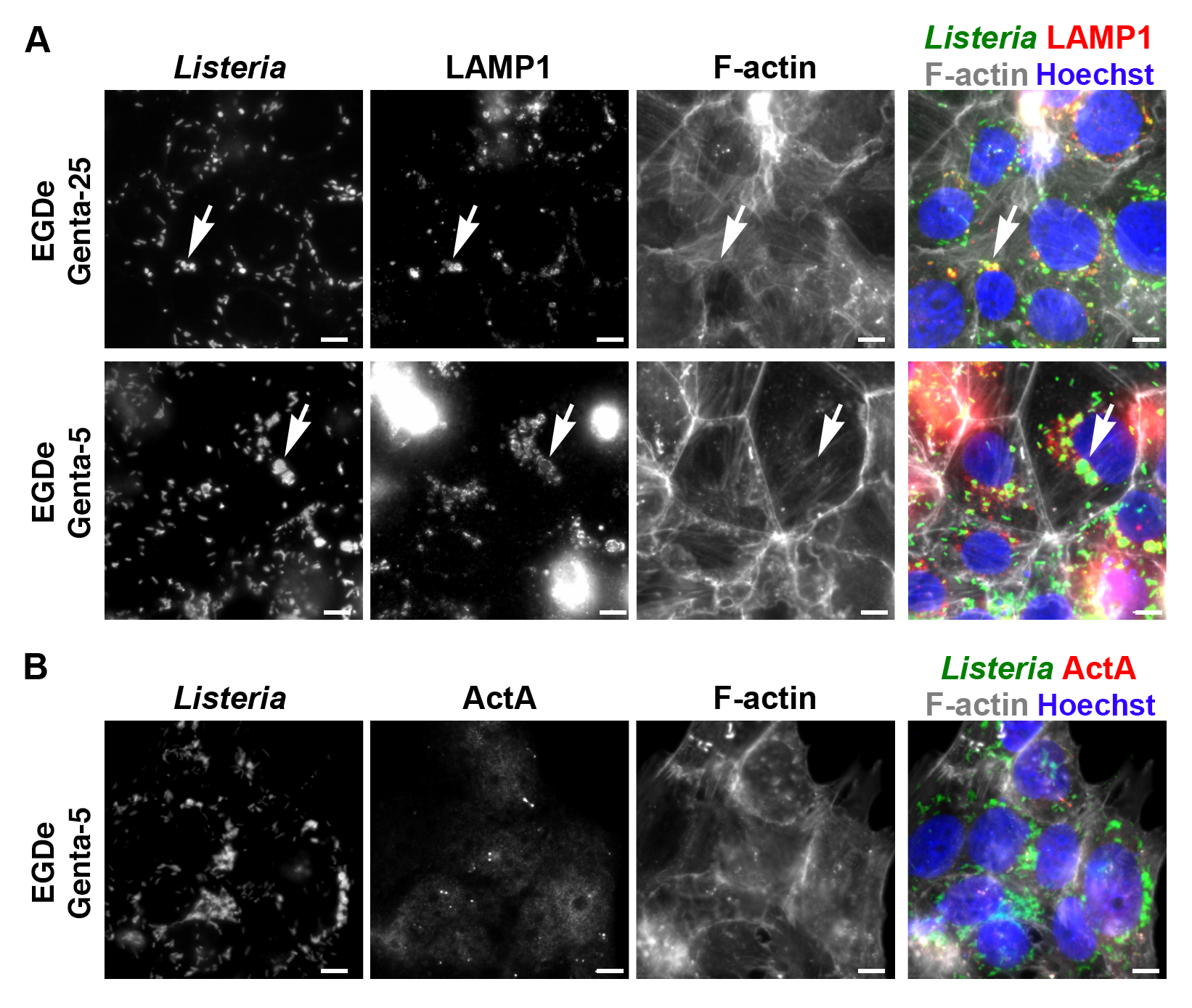

Supplement: S11 Fig — Representative micrographs of JEG3 cells infected with L. monocytogenes EGDe (MOI ~ 0.1; without 10-min exposure to gentamicin 100 μg/mL) for 72h in presence of gentamicin 5 or 25 μg/mL. Cells were labeled with Listeria polyclonal antibodies (green in the overlay), LAMP1 (A) or ActA (B) monoclonal antibodies (red in the overlay) and Hoechst (Blue in the overlay). White arrows point LisCVs. Bar: 10 μm. (TIF) [file ppat.1006734.s011.tif]

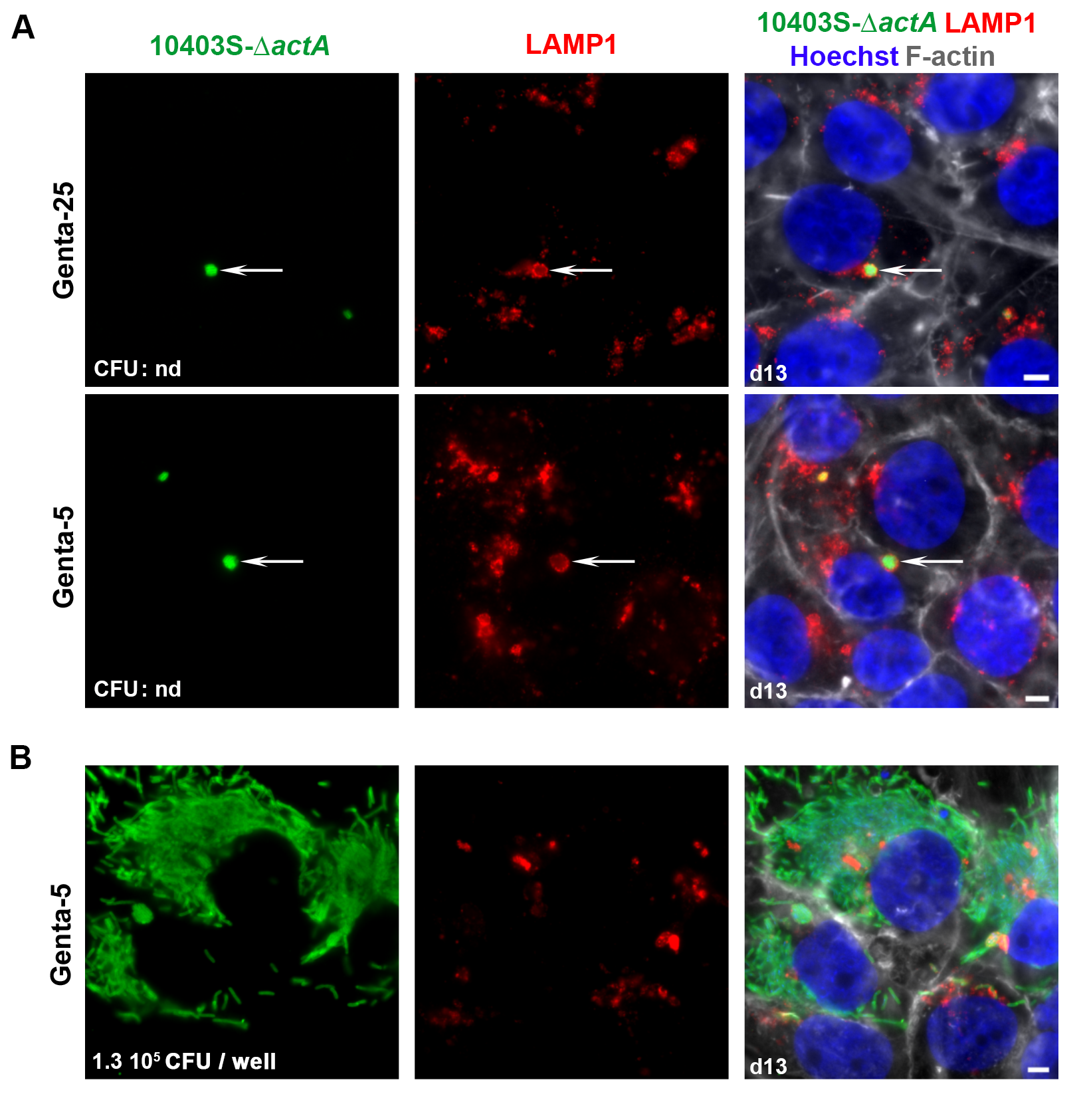

Supplement: S12 Fig — JEG3 cells infected with L. monocytogenes 10403S-ΔactA (MOI ~ 1) and propagated up to d13 in presence of gentamicin 5 or 25 μg/mL. A 12 mm coverslip placed in the well was recovered just before lysing the cells, in order to process the same cells for immunofluorescence and CFU counts. Micrographs show representative cells from (A) wells leading to VBNC bacteria (not detectable colony, “nd”) or (B) bacteria forming colonies (1.3 105 CFU/well). Cells were labeled with Listeria polyclonal antibodies (green), LAMP1 monoclonal antibodies (red), fluorescent phalloidin (white) and Hoechst (Blue). White arrows point LAMP1+ bacteria. Bar: 5 μm. (TIF) [file ppat.1006734.s012.tif]
